# Identification of miRNAs involved in fruit ripening by deep sequencing of *Olea europaea* L. transcriptome

**DOI:** 10.1371/journal.pone.0221460

**Published:** 2019-08-22

**Authors:** Fabrizio Carbone, Leonardo Bruno, Gaetano Perrotta, Maria B. Bitonti, Innocenzo Muzzalupo, Adriana Chiappetta

**Affiliations:** 1 Department of Biology, Ecology and Earth Science, University of Calabria, Arcavacata Rende (CS) IT; 2 Research Centre for Olive, Citrus and Tree Fruit—Council for Agricultural Research and Economics, Rende (CS) IT; 3 ENEA, TRISAIA Research Center, S.S. Jonica, Rotondella (MT) IT; Wuhan Botanical Garden, CHINA

## Abstract

**Background:**

The ripening process of olive fruits is associated with chemical and/or enzymatic specific transformations, making them particularly attractive to animals and humans. In olive drupes, including ‘Cassanese’ ones, ripening is usually accompanied by progressive chromatic change, resulting in a final red-brown colourization of both epidermis and mesocarp. This event has an exception in the ‘Leucocarpa’, in which we observed the destabilization in the equilibrium between the chlorophyll metabolism and that of the other pigments, particularly the anthocyanins, whose switch-off during maturation promotes the white colouration of the fruits. Recently, transcription profiling of ‘Leucocarpa’ and ‘Cassanese’ olives along ripening, performed through an Illumina RNA-seq approach, has provided useful insights on genes functions involved in fruit maturation such as those related to the biosynthesis of flavonoids and anthocyanins.

**Methodology:**

To assess expression alterations of genes involved in flavonoids and anthocyanins biosynthetic pathways during ripening, possibly caused by small nuclear RNA (snRNA) in olive drupes, snRNA libraries from ‘Leucocarpa’ and ‘Cassanese’ were constructed with RNAs extracted at 100 and 130 Days After Flowering (DAF) and sequenced by an Illumina approach. 130 conserved microRNAs (miRNA) in the *Viridiplantae* belonging to 14 miRNA families were identified. Regarding the 130 conserved miRNAs, approximately the 48% were identified in all libraries, 5 and 18 miRNAs were shared between the “Cassanese” (C100, C130) and “Leucocarpa” (L100, L130) libraries, respectively.

**Conclusion:**

For the remaining reads not-matching with known miRNAs in the *Viridiplantae*, we combined secondary structure and minimum free energy to discover novel olive miRNAs. Based on these analyses, 492 sequences were considered as putative novel miRNAs. The putative target genes of identified miRNA were computationally predicted by alignment with the olive drupe transcripts obtained from the same samples. A total of 218 transcripts were predicted as targets of 130 known and 492 putative novel miRNAs. Interestingly, some identified target genes are involved in negative regulation of anthocyanin metabolic process. Quantification of the expression pattern of three miRNA and their target transcripts by qRT-PCR assay confirmed the results of Illumina sequencing.

## Introduction

The snRNAs are a class of non-coding RNAs, very abundant in the eukaryotic genome, and include the small interfering RNAs (siRNAs) and microRNAs (miRNAs). The miRNA when expressed, match with the 3’untranslated region (UTR) of target mRNAs determining gene silencing through the transcript cleavage or the translational inhibition [1–5]. In plants RNAs molecules of ~ 25 nt in length have been associated with posttranscriptional gene silencing [6–9].

The RNA silencing has been proposed as a viral and tranposons defense mechanism [9], but since the discover of miRNAs in the worm *Caenorhabditis elegans*, as molecules controlling the timing of the larval development [10–11], it was suggested that they can be used by organisms for the regulation of endogenous genes. Currently, an increased number of studies established their role as key regulators of genome expression and their involvement in the modulation of pivotal events in development and response to environmental cues in most eukariotes. In plants, the importance of miRNAs for development is supported by the abnormalities observed in mutants and transgenic lines showing defects in miRNA accumulation or activity [12–17].

So far, plant miRNAs have been experimentally analysed and bioinformatically predicted in many relevant crop species, including *Pyrus bretschneideri* [18], *Citrus sinensis* [19], *Solanum lycopersicum* [20] and *Olea europaea* [21, 22]. Such studies revealed miRNAs to be master regulators, targeting TFs involved in diverse morpho-physiological processes including fruit development, ripening, and fruit alternate bearing [20, 21, 23, 24]. Accordingly, miR166 and miR168 are highly expressed in cotton ovules [25], miR168 is involved in developmental process of tomato fleshy fruits and seeds [26, 27] and miR169, suppresses C class MADS box genes in relation to carpel development suggesting that miR169 is involved in fruit development [28]. Moreover, in tomato fruit, miR157 controls Colourless Non-Ripening (*CNR)* expression which in turn modulate ripening-related gene expression [29] and carotenoid biosynthesis [24, 30]. miR159 negatively regulates MYB TFs which are important in seed germination and flower development [31]. Interestingly, in recent years, various reports suggested a significant role of miRNAs in regulating the biosynthesis and accumulation of secondary metabolites in plants [32, 33].

Anthocyanins are among the most relevant classes of secondary metabolites which commonly accumulate in flowers and fruits, but are also present in leaves, stems, and storage organs [34–37]. In addition, they can be used as dietary nutraceutics, exerting benefits for human health [37–40].

The anthocyanins are synthesized through the phenylpropanoid pathway, one of the most extensively studied pathways of secondary metabolites for transcriptional regulation in plants [41–43].

Recently, scientific efforts have been directed toward understanding the involvement of miRNAs in the post-transcriptional regulation of the phenylpropanoid pathway. It has been demonstrated that the miR156 targeted the SQUAMOSA PROMOTER BINDING PROTEIN- LIKE 9 (SPL9) TF, involved in both the dihydroflavonols and leucoanthocyanidin synthesis [33]. This occurs through the destabilization by SPL9 of the regulatory complex MYB-bHLH-WD40, that controls polyphenolic biosynthetic steps [44–50] and interferes with the *DFR* expression. Besides, in *Arabidopsis* SPL9 inhibits the expression of anthocyanin biosynthetic genes [51, 52]. Accordingly, higher levels of miR156 reduce the SPL activity and enhance the expression of F3H, DFR, and other anthocyanin biosynthetic-related genes [33, 51]. On the other hands, high levels of SPL reduce the accumulation of anthocyanins and increases flavonols production [33, 51].

Anthocyanin compounds are among the secondary metabolites which accumulate in olive fruit, together with other polyphenolic compounds [53]. Therefore olive (*Olea europaea* L. subsp. *europaea* var. *europaea*) drupes and olive oil represent an interesting source of phytochemicals, that have beneficial effects on health. Interestingly, several epidemiological studies suggest that the intake of olives and olive oils are responsible for the reduction of the incidence of cardiovascular diseases, certain types of cancer, and aging-related pathologies [54]. Anyway, the relative concentration of the biologically active compounds with health attributions in these food matrices is closely dependent on several factors, varying in relation to environmental growth conditions, the genetic traits, the varieties as well as the ripeness stage [55, 56].

Recently, transcription profiling performed through an Illumina RNA-seq approach on ‘Leucocarpa’ and ‘Cassanese’ drupes collected at different ripening stage, allowed us to obtain some information about genes involved in maturation such as genes involved in the biosynthetic pathways of flavonoid and anthocyanin [57]. In particular, we identified some TF members with similarity to MYB, MIC and WD40 family directly related to anthocyanin accumulation. Interestingly the transcript levels of these TFs were higher in Cassanese cv than in ‘Leucocarpa’, which is an olive variety characterized by a switch-off in skin colour at full ripeness.

Based on the participation of multiple miRNAs in controlling fruit ripening, in the present study we aimed to identify specific miRNAs involved in flavonoid and anthocyanin metabolism in olive drupes using high-throughput sequencing combined with bioinformatics analysis and molecular experiments. Then, four snRNA libraries covering two developmental stages of fruit ripening of ‘Leucocarpa’ and ‘Cassanese’ were constructed with RNAs from drupes collected at 100 and 130 Days After Flowering (DAF) and sequenced by an Illumina approach.

## Material and methods

### Plant material

Olives (*Olea europaea* L.) of two varieties Cassanese and Leucocarpa (S1 Fig), were harvested from trees grown under natural conditions in the field germplasm collection of the CREA—Research Centre for Olive, Citrus and Tree Fruit (Rende, Cosenza, Italy, latitude 39°21′57′′N, longitude 16°13′44′′ E and altitude 210 masl). Ripening drupes (n = 30) with 100 and 130 DAF were randomly collected from 20-years-old plants, fixed in liquid nitrogen and stored at -80°C. For quantitative real-time PCR (qRT-PCR) we used the same samples managed in a similar way but collected four years later at the same DAF.

### Small RNA library preparation and sequencing

Total RNA was isolated from the epi-mesocarp tissues of drupes with the RNeasy Plant Mini Kit (Qiagen, Hilden, Germany) and treated with DNase I (Roche, Milan Italy), as previously described [57]. Agilent 2100 bioanalyzer (Agilent technologies) assay was carried out to evaluate RNA quality and quantity. We used for cDNA library preparation only the samples isolated and purified with a concentration of ≥ 400 ng/μl, OD260/280 = 1.8 ~ 2.2, RNA 28S:18S ≥ 1.0 and RNA Integrity Number (RIN) ≥ 7.0. Library preparation and sequencing was carried out by Technology Services of the Institute of Applied Genomics (IGA, Udine, Italy). Upon the quality control of the starting material libraries were generated and size was selected in a range maximizing the number of relevant reads. Sequencing was performed on HiSeq2500 in a 50 bp single-read mode.

### miRNA analysis

Raw reads were pre-processed following pipeline illustrated as a flow diagram in S2 Fig. Briefly, pre-processing was based on miRExpress software [58] to merge the identical reads into a unique read, to count each unique read and to remove full and partial adaptor sequences. Sequences below 18 bp and above 32 bp were also discarded.

In order to identify known miRNAs in olive, the remaining unique sequences were aligned against known *Viridiplantae* miRNA (miRBase database version 21.0) [59] by using miRExpress software.

To identify putative novel miRNAs, sequences that no matched the miRBase database were aligned with genome sequence of the olive tree [60] using the SOAP 2.0 software [61]. The secondary structures of the matched sequences were analysed by the Mireap software (http://sourceforge.net/projects/mireap/) in order to identify new candidate miRNAs according to the following parameters: (1) miRNA sequence length 18–32 bp, (2) miRNA reference sequence length 18–32 bp, (3) maximal copy number of miRNAs on reference 600, (4) maximal free energy allowed for a miRNA precursor -30 kcal/mol, (5) maximal space between miRNA 450 bp, (6) minimal base pairs of miRNA 16bp, (7) maximal bulge of miRNA 3bp, (8) maximal asymmetry of duplex 4 bp, (9) flank sequence length of miRNA precursor 20 bp. The selected sequences were then folded into a secondary structure by Mfold program [62].

The frequency of both known and putative novel miRNA read count was normalized as transcript per million (TPM) and normalization of miRNA expression levels among four libraries was carried out as reported by [63].

miRNA target identification was carried out by alignment with the olive drupe transcripts obtained through an Illumina RNA-seq approach [57] by Bowtie program [64]. Target genes of both conserved and novel miRNAs were compared with *Arabidopsis* protein database and Gene Ontology (GO) and KEGG pathway enrichment analyses were performed using KOBAS 3.0 [65]. The enrichment analysis of each GO and KEGG term was performed using the hypergeometric test (Fisher's exact test), considering transcriptome obtained through an Illumina RNA-seq approach as background ‘genome’. Then, any GO and KEGG terms with an FDR adjusted p-value (q-value) less than of 0.05 were considered as the enriched ones.

Venn diagram was generated by a web-tool (http://bioinformatics.psb.ugent.be/webtools/Venn/) and part of miRNA expression data was visualized in a circular layout by Circos Table Viewer v0.63–9 [66].

The data discussed in this publication have been deposited in NCBI's Gene Expression Omnibus [67] and are accessible through GEO Series accession number GSE104763 (https://www.ncbi.nlm.nih.gov/geo/query/acc.cgi?acc=GSE104763).

### Stem-loop quantitative real-time PCR

For stem-loop quantitative real-time PCR (qRT-PCR) reverse transcription reactions were performed as described previously with some modification [21]. Each reaction solution (final volume 10 μl) contained 1 μg of total RNA, 1 μM RT stem-loop primer (S1 Table) and Superscript III (Invitrogen), according to manufacturer’s instructions. The qRT-PCR was carried out using Applied Biosystems® 7500 fast instrument and a Thermo Scientific Maxima SYBR Green/ROX qPCR Master Mix (2X) according to manufacturer’s instructions. Each RT product was amplified with universal reverse primer and three specific miRNA forward primers (S1 Table). Three independent biological replicates were used for each cDNA and miRNA. PCR conditions were: 15ʹ at 95°C followed by 40 cycles of incubations at 95°C × 5ʹʹ, at 56°C × 10ʹʹ and at 72°C × 60ʹʹ. At the end of the PCR, the melting temperature of the amplicons was measured by denaturing the amplified DNA, in order to determine whether the amplified products are homogeneous and the correct product has been specifically amplified.

### Quantitative real-time PCR of predicted target genes

First strand cDNA was synthesized from 1 μg total RNA with oligo-d(T) and Superscript III (Invitrogen), according to the manufacturer’s instructions. 50 ng cDNA treated with RNase was used as template for qRT-PCR assays, carried out using Applied Biosystems® 7500 fast instrument and a Thermo Scientific Maxima SYBR Green/ROX qPCR Master Mix (2X) according to manufacturer’s instructions and with primers for target transcripts of miR168, miR166 and miR159 and for elongation factor 1-alpha (ef-1a). Three independent biological replicates were used for each cDNA and transcript. PCR conditions were: 95°C × 5ʹʹ, followed by 45 cycles at 95°C × 15ʹʹ and at 58°C × 60ʹʹ. At the end of the PCR, the melting temperature of the amplicons was measured by denaturing the amplified DNA, in order to determine whether the amplified products are homogeneous and the correct product has been specifically amplified. Relative template abundance was quantified using the standard curve method, normalizing each data against the quantity of the actin transcript [68]. Serial 10-fold dilutions of each gene amplicon were used to calculate the amplification efficiency for each target and control transcript.

## Results

To investigate the effects of small RNAs (sRNA) during fruit development in *Olea europaea* and identify potential novel miRNAs, we performed a deep sequencing of the sRNA libraries resulting from ripening fruits of two different olive cultivars. Drupes were collected at 100 and 130 DAF of both Leucocarpa (L) and Cassanese (C) cultivars and were used to generate four distinct sRNA libraries.

**Fig 1:**
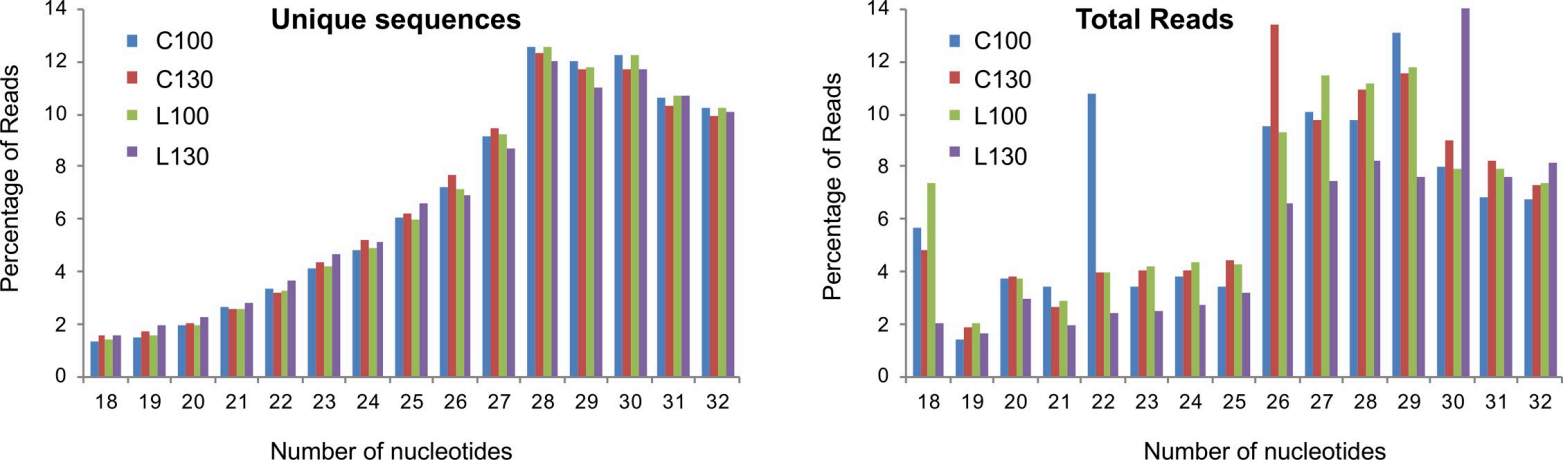
Small RNAs in each of the four libraries. Length (bp) distribution of unique and total sequencing reads. Percentage of reads in specific length per library to the summed reads in corresponding library was shown.

Illumina deep sequencing generated a total of 171,086,524 raw reads. After processing of primary reads by removing adaptors and discarding low quality reads, 2,488,061, 7,171,566, 3,106,156 and 3,901,395 total filtered reads were counted for C100, C130, L100 and L130 libraries, respectively (Table 1). The length of the sRNAs varied from 18 to 32 bp, being 28 bp the most abundant read length (Fig 1). Furthermore, these 16,667,178 clean reads were represented by 2,060,862 unique tags; 143 unique tags were sequenced more than 10k times, whereas 77.3% and 11.9% were present only one and two times, respectively.

**Table 1:** Summary of sequencing results from two varieties (ʹLeucocarpaʹ and ʹCassaneseʹ) at the two different ripening stages (100 and 130 DAF).

	C100		C130		L100		L130	
	Total	Unique	Total	Unique	Total	Unique	Total	Unique
Raw data	26,513,964	7,048,949	70,190,746	13,462,730	34,379,362	8,167,774	40,002,452	5,480,276
Adapter removed	4,006,593	985,199	11,863,144	2,593,282	5,356,148	1,370,116	5,777,137	1,043,617
Filtered data	2,488,061	339,875	7,171,566	888,038	3,106,156	455,013	3,901,395	377,936

### Identification of conserved miRNAs

To identify miRNAs from ripening drupes, the sequenced sRNA fragments were aligned against miRBase database (version 21.0) that includes 8,496 known miRNAs belonging to 73 plant species.

Comparison analyses revealed 130 conserved miRNAs (261,069 reads) clustered in 14 families; reduced to 11 and 10 mRNA families when limiting the dataset to Cassanese and Leucocarpa cv, respectively (S2 Table).

8 out of the 14 identified miRNA groups were already reported in olive [21, 69] while the remaining 6 are newly discovered (miR3630, miR5083, miR5538, miR6300, miR845 and miR894).

**Fig 2:**
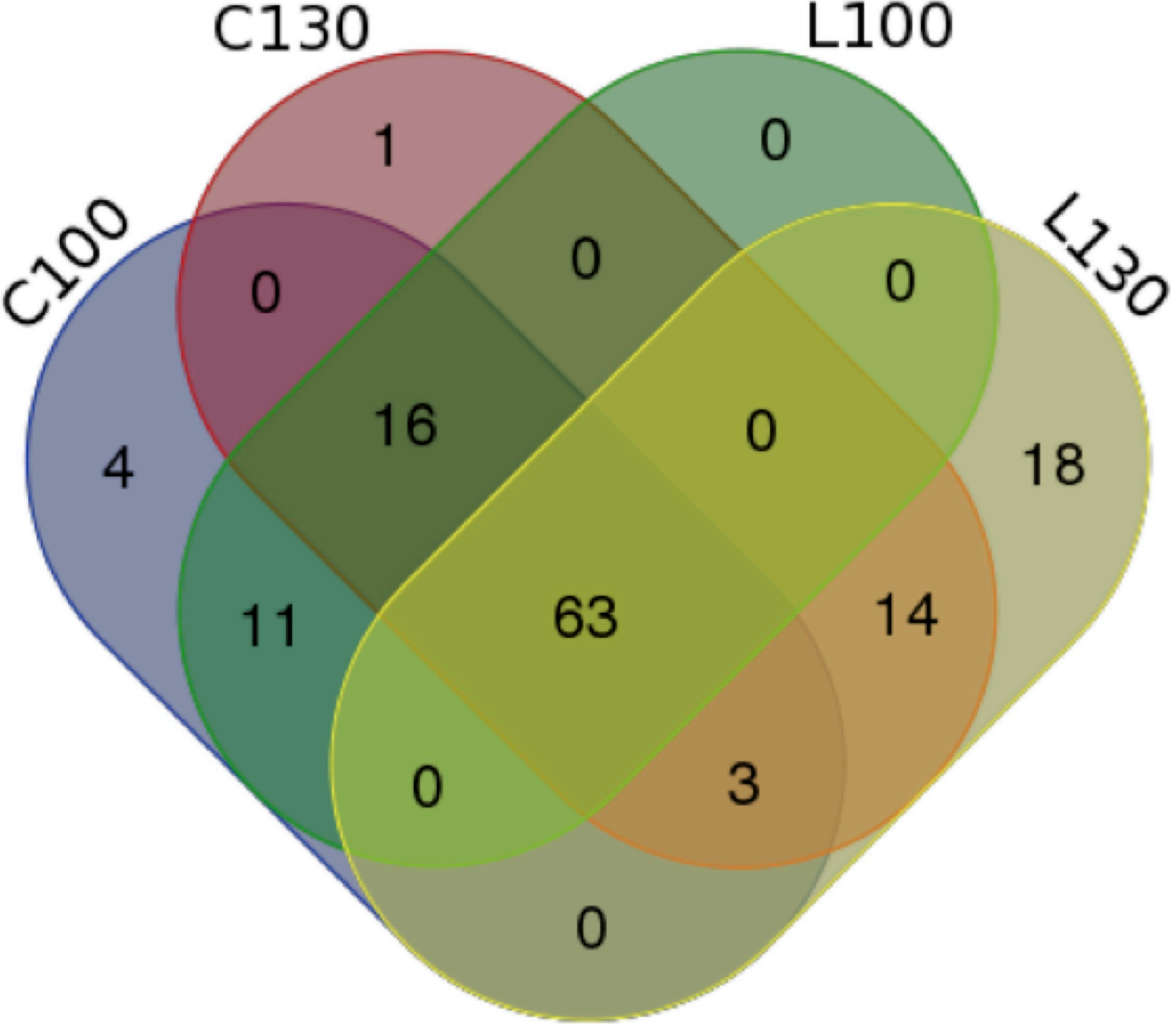
Venn diagram of the conserved miRNA in the four libraries. The intersecting portions of the Venn diagrams reports the number of common conserved miRNA families among the different comparisons between the two varieties (ʹLeucocarpaʹ and ʹCassaneseʹ) at the two different ripening stages (100 and 130 DAF).

About half of the identified miRNAs (63) were identified in all libraries, but only 5 and 18 miRNAs are shared within ʹCassaneseʹ and ʹLeucocarpaʹ samples respectively, regardless to the ripening stage (Fig 2). Interestingly, a small but significant number of miRNAs, spanning around 8–11% of the total, are expressed at a specific ripening stage (100 DAF or 130 DAF), instead their expression is not subjected to relevant intra-species variability. Hence, they appear to be exclusively required at different times during the fruit ripening of olive (Fig 2).

The overall diversity of miRNA distribution and among the sequenced samples is quite constant in 3 out of the 4 libraries. ʹLeucocarpaʹ ripe library (L130), instead, contains the higher number of exclusively present (18) or absent (16) miRNA species (Fig 2).

The abundance of defined miRNA was determined by measuring the normalized frequency of matching reads in the sequenced dataset. At a general level, this revealed that, most of the identified miRNA families were represented at low frequencies. On the other hand, higher redundancy was confined to only 4 families—miR166, miR159, miR168, miR156 - (S2 Table).

**Fig 3:**
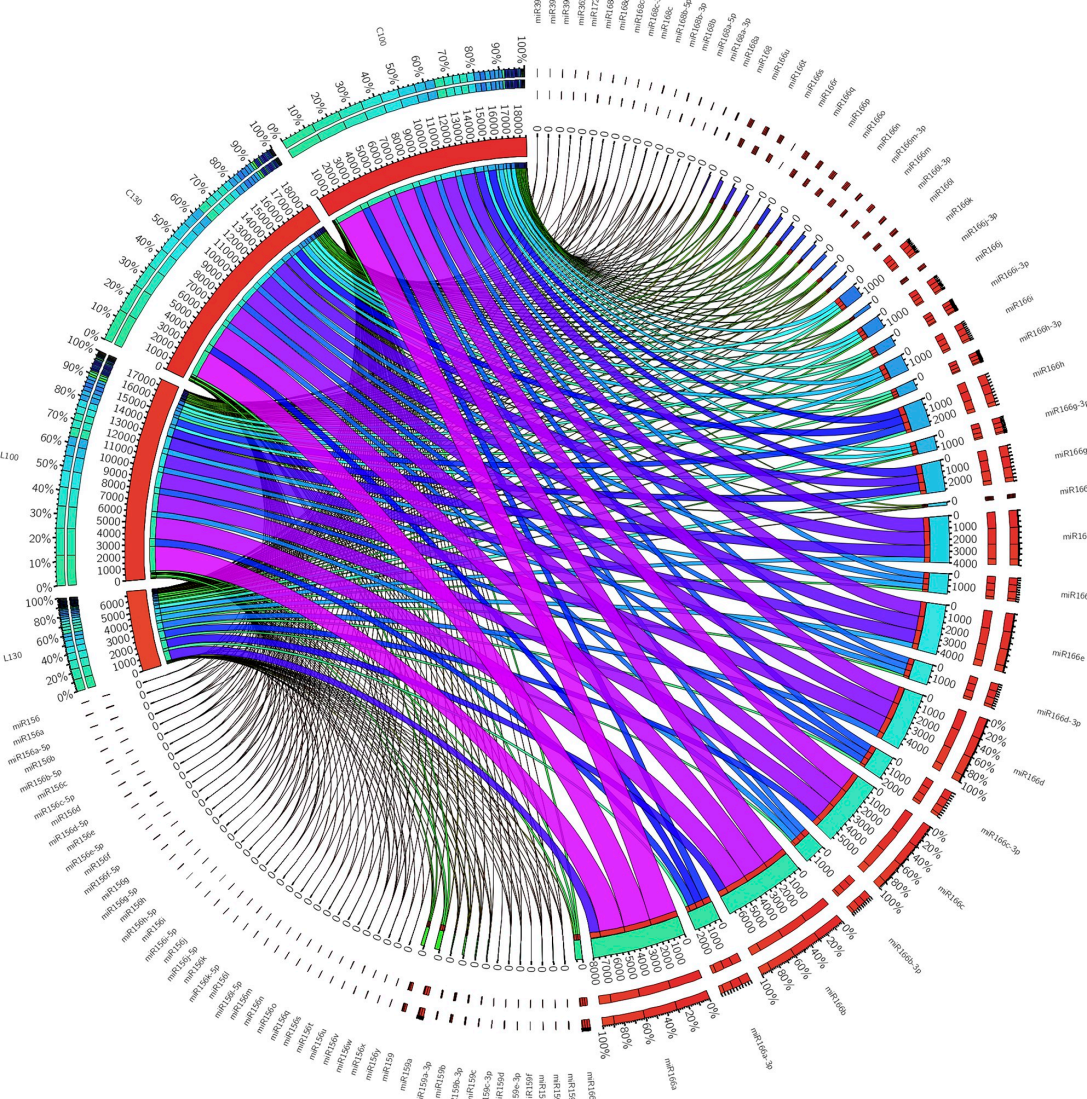
Distribution of expression of the 130 conserved miRNAs belong the four libraries. The data were visualized via Circos software [66]. The width of bars from each library and miRNA indicates their relative abundance.

The miR166 family (59,825 reads and 34 variants) showed an expression 2.7-fold higher in ʹCassaneseʹ than in the Leucocarpa cv during 100–130 DAF transition (C130 *vs* L130). Furthermore, miRNA159 (1,204 reads) and miR156 (201 reads) families, with 13 and 36 variants in the dataset, were 2.7- and 2.3-fold higher in C130 with respect to L130. Finally, the miR168 family (322 reads and 11 variants), was more expressed in Cassanese cv, 4.5- and 1.5- fold in unripe and ripe drupes, respectively (Fig 3), suggesting a potential key role of these miRNAs in affecting gene expression of the white olive ʹLeucocarpaʹ.

Additional expression alterations were also revealed at the single miRNA species. In fact, L130 showed the lowest accumulation of miRNAs, with 63 downregulated miRNAs species, with respect to the other samples.

### Prediction of novel olive miRNAs

**Fig 4:**
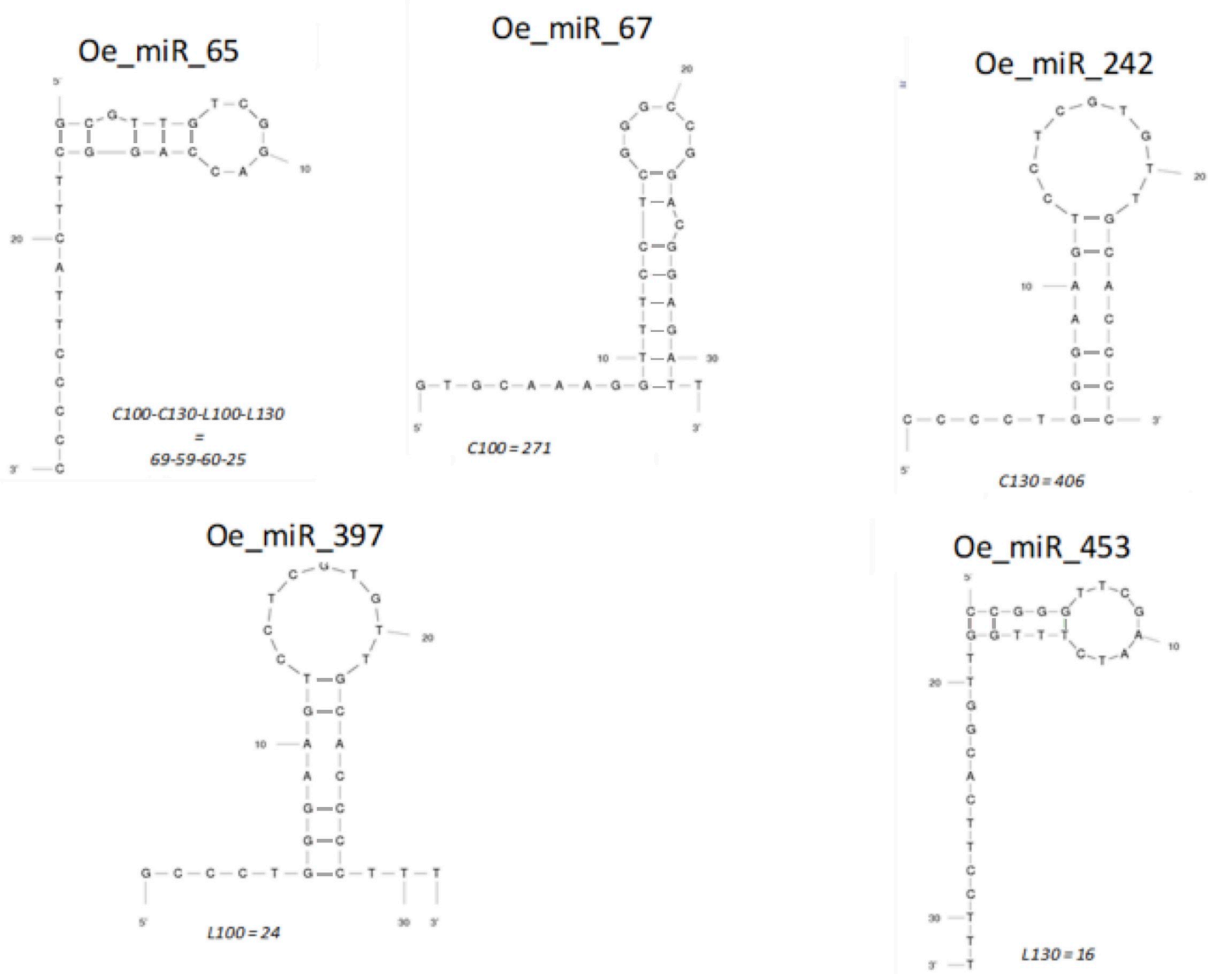
Predicted secondary hairpin structures of some novel miRNAs identified in this study by mFold.

**Fig 5:**
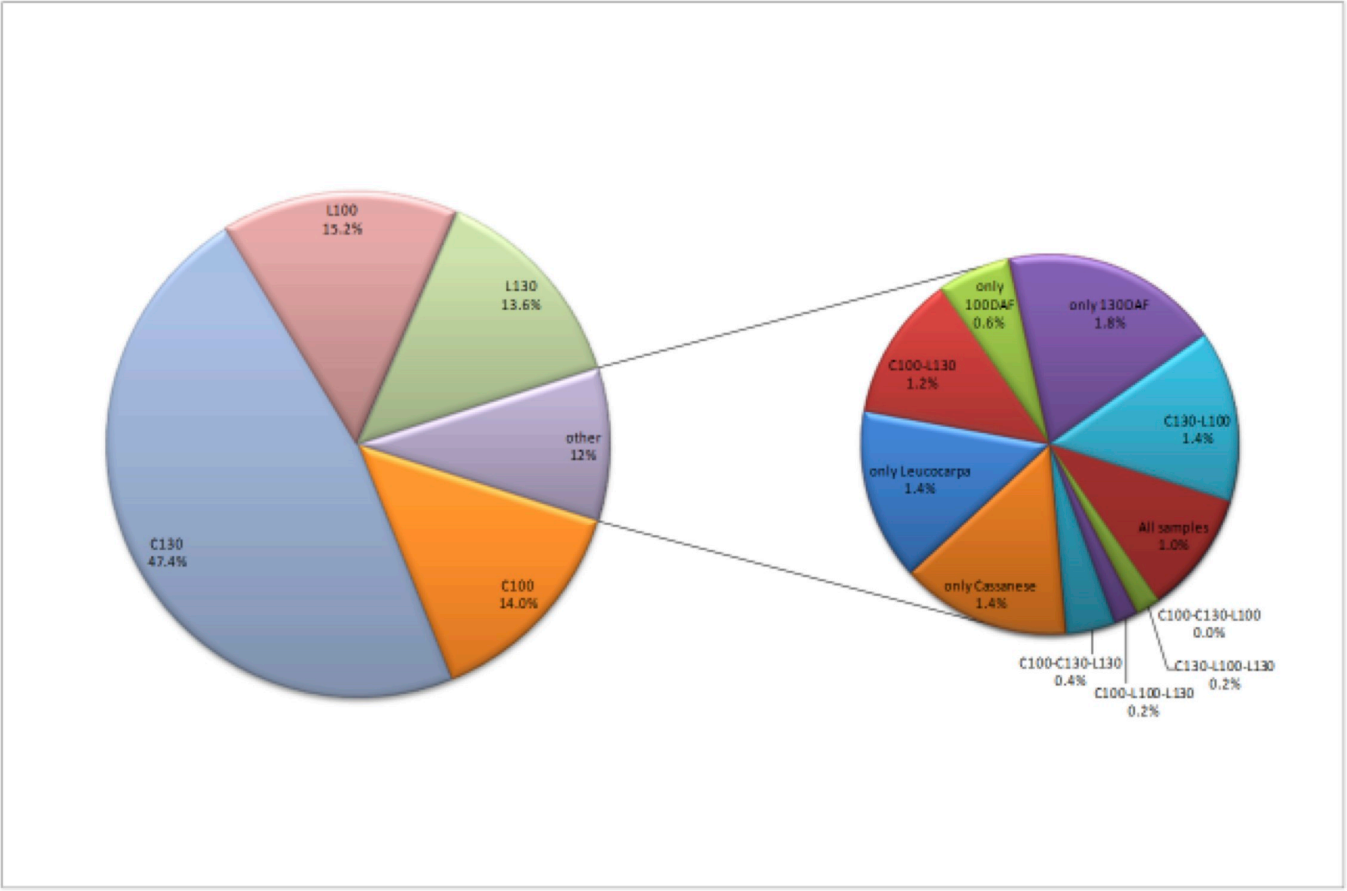
Summary of common and specific identified novel miRNAs among different libraries.

For the remaining reads not-matched with known *Viridiplantae* miRNAs, once mapped to genome sequence of the olive tree [60], we combined secondary structure and minimum free energy to discover novel miRNAs in olive. Based on these analyses, 11,519 novel miRNA-like reads were identified from the 4 sequenced libraries, representing 492 unique miRNAs (S3 Table). The folded secondary structures of the putative novel miRNAs are shown in Fig 4 and some of them showed peculiar expression profiles (S3 Table, Fig 5). Thus, 149 were expressed only in libraries created from ʹLeucocarpaʹ genotype. Some of them (Oe_mir_355, Oe_mir_368, Oe_mir_379, Oe_mir_380, Oe_mir_398, Oe_mir_408, Oe_mir_416) were detected in both 100 and 130 DAF libraries (L100, L130), while 75 and 67 were found only in the unripe fruit library (L100) and in the ripe fruit library (L130), respectively. On the contrary, 309 putative novel miRNAs were specifically detected in ʹCassaneseʹ libraries. Some of them (Oe_mir_5, Oe_mir_19, Oe_mir_23, Oe_mir_29, Oe_mir_33, Oe_mir_37, Oe_mir_43) were detected in both 100 and 130 DAF (C100, C130), while 69 and 233 were found only in the unripe fruit library (C100) and in the ripe fruit library (C130), respectively (S3 Table, Fig 5). Interestingly, of the putative novel miRNAs exclusively expressed in the Cassanese cv, Oe_mir_242 in ripe library and Oe_mir_67 showed an elevated read frequency in unripe library (406 and 271 reads) (Fig 4). Of the 147 putative novel miRNA expressed only in unripe libraries, only some of them (Oe_mir_54, Oe_mir_63, Oe_mir_70) were detected in both varieties (L100, C100), while 69 and 75 were specifically found in Cassanese and Leucocarpa varieties (C100, L100), respectively. On the contrary, 309 putative novel miRNA were detected only in ripe fruit libraries. 9 of them (Oe_mir_120, Oe_mir_184, Oe_mir_198, Oe_mir_226, Oe_mir_265, Oe_mir_303, Oe_mir_304, Oe_mir_329, Oe_mir_340) were identified in both genotype libraries (L130,C130), while 233 and 67 were found only in Cassanese and Leucocarpa varieties (C130, L130), respectively (S3 Table, Fig 5).

Finally, Oe_mir_32, Oe_mir_46, Oe_mir_49, Oe_mir_65, Oe_mir_91 were detected in all samples. Two of them (Oe_mir_49, Oe_mir_65) showed a similar expression pattern with a decrement of read frequencies during the ripening. Of the rest three, for Oe_mir_32 and Oe_mir_46 were observed an opposite pattern between genotypes with an increment and a decrement of read frequencies during 100–130 DAF transition in ʹCassaneseʹ and in ʹLeucocarpaʹ, respectively.

### Target prediction of olive miRNAs

**Fig 6:**
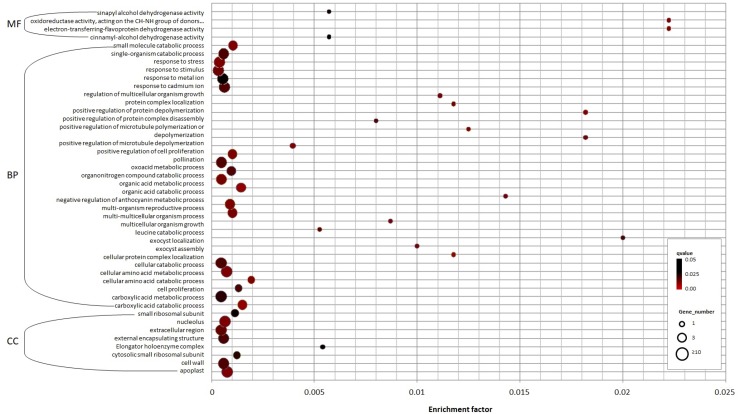
Gene Ontology (GO) of unigenes targeted by conserved and predicted miRNAs. The significantly enriched GO terms were categorized according to biological process, molecular function and cellular component. The enrichment factor (total number of the specific GO terms in the predicted miRNA targets vs. total number of the specific GO term in the reference olive trascriptome) is on the x-axis. The size of each point represents the number of genes enriched in a particular GO term. A larger enrichment factor value and lower Q-values indicates a greater degree of enrichment.

To increase insights into the functions of known and putative novel miRNAs in olive, putative target genes were computationally predicted by alignment with the olive drupe transcripts previously obtained from the same samples [57]. A total of 32 and 186 transcripts were predicted as targets of 130 known and 492 putative novel miRNA, respectively. All olive cDNAs predicted to be targets of known miRNAs were orthologues of miRNA target gene in *Coffea canephora* and other plant species (Table 2). The identified target genes are involved in a broad range of biological processes. In particular, GO enrichment analysis revealed that the target genes of olive miRNAs appeared to be significantly enriched in 44 GO terms (q-value < 0.05), the majority of which regrouped in biological process (72.7%) category followed by cellular component (18.2%) and molecular function (9.1%) (Fig 6, Table 2, S4 Table). Many target genes were related to ʹcatabolic processʹ (GO:0046395, GO:0016054, GO:0009063, GO:0044282, GO:1901565, GO:0044248, GO:0044712, GO:0006552) followed by process required for the high protein turnover existing in the ripening fruit as ʹribosomal proteinʹ, ʹprotein depolymerization and polymerizationʹ (GO:0031117, GO:1901881, GO:0031112, GO:0008284, GO:0034629, GO:0031503, GO:0008283, GO:0005618, GO:0022627, GO:0015935) and by ʹphenylpropanoid regulationʹ (GO:0031538, GO:0045551, GO:0052747). Among all, two molecular functions involved in oxidative phosphorylation (GO:0016649 and GO:0004174) shown a more enrichment factor (total number of the specific GO terms in the predicted miRNA targets vs. total number of the specific GO term in the olive transcriptome) (Fig 6, S4 Table).

**Table 2:** Predicted olive cDNA targets for known olive miRNAs.

	Functional Annotation			
miRNA	RNA_seq_ID	NCBI_ID	Gene ontology (GO)	KEGG pathway
miR156	comp75091_c0_seq1	GO:0045551	cinnamyl-alcohol dehydrogenase activity	Biosynthesis of secondary metabolites
		GO:0050896	response to stimulus	Phenylpropanoid biosynthesis
		GO:0052747	sinapyl alcohol dehydrogenase activity	Metabolic pathways
	comp75090_c0_seq1	GO:0045551	cinnamyl-alcohol dehydrogenase activity	Metabolic pathways
		GO:0050896	response to stimulus	Phenylpropanoid biosynthesis
		GO:0052747	sinapyl alcohol dehydrogenase activity	Biosynthesis of secondary metabolites
	comp42353_c0_seq1			
miR159	comp19510_c0_seq1	GO:0004174	electron-transferring-flavoprotein dehydrogenase activity	
		GO:0006082	organic acid metabolic process	
		GO:0006520	cellular amino acid metabolic process	
		GO:0006552	leucine catabolic process	
		GO:0009063	cellular amino acid catabolic process	
		GO:0016054	organic acid catabolic process	
		GO:0016649	oxidoreductase activity, acting on the CH-NH group of donors, quinone or similar compound as acceptor	
		GO:0019752	carboxylic acid metabolic process	
		GO:0043436	oxoacid metabolic process	
		GO:0044248	cellular catabolic process	
		GO:0044282	small molecule catabolic process	
		GO:0044712	single-organism catabolic process	
		GO:0046395	carboxylic acid catabolic process	
		GO:0050896	response to stimulus	
		GO:1901565	organonitrogen compound catabolic process	
	comp13723_c0_seq1			
	comp33358_c0_seq1	GO:0004174	electron-transferring-flavoprotein dehydrogenase activity	
		GO:0006082	organic acid metabolic process	
		GO:0006520	cellular amino acid metabolic process	
		GO:0006552	leucine catabolic process	
		GO:0009063	cellular amino acid catabolic process	
		GO:0016054	organic acid catabolic process	
		GO:0016649	oxidoreductase activity, acting on the CH-NH group of donors, quinone or similar compound as acceptor	
		GO:0019752	carboxylic acid metabolic process	
		GO:0043436	oxoacid metabolic process	
		GO:0044248	cellular catabolic process	
		GO:0044282	small molecule catabolic process	
		GO:0044712	single-organism catabolic process	
		GO:0046395	carboxylic acid catabolic process	
		GO:0050896	response to stimulus	
		GO:1901565	organonitrogen compound catabolic process	
	comp46826_c0_seq1			
	comp68814_c0_seq1	GO:0006950	response to stress	
		GO:0050896	response to stimulus	
miR166	comp97992_c0_seq1	GO:0005576	extracellular region	Sphingolipid metabolism
		GO:0005618	cell wall	Glycosphingolipid biosynthesis—globo series
		GO:0030312	external encapsulating structure	Galactose metabolism
		GO:0044248	cellular catabolic process	Glycerolipid metabolism
		GO:0044712	single-organism catabolic process	
		GO:0048046	Apoplast	
		GO:1901565	organonitrogen compound catabolic process	
	comp56340_c0_seq1	GO:0005730	Nucleolus	Spliceosome
		GO:0006950	response to stress	
		GO:0050896	response to stimulus	
	comp62505_c0_seq1	GO:0008283	cell proliferation	
		GO:0008284	positive regulation of cell proliferation	
		GO:0050896	response to stimulus	
miR168	comp9440_c0_seq1	GO:0006950	response to stress	Base excision repair
		GO:0050896	response to stimulus	Purine metabolism
				Pyrimidine metabolism
				Homologous recombination
				Metabolic pathways
				DNA replication
				Nucleotide excision repair
	comp55643_c0_seq1			Alanine, aspartate and glutamate metabolism
	comp60984_c0_seq1	GO:0006950	response to stress	
		GO:0008283	cell proliferation	
		GO:0008284	positive regulation of cell proliferation	
		GO:0031538	negative regulation of anthocyanin metabolic process	
		GO:0033588	Elongator holoenzyme complex	
		GO:0050896	response to stimulus	
	comp9240_c0_seq1			
	comp17765_c0_seq1	GO:0006950	response to stress	
		GO:0008283	cell proliferation	
		GO:0008284	positive regulation of cell proliferation	
		GO:0031538	negative regulation of anthocyanin metabolic process	
		GO:0033588	Elongator holoenzyme complex	
		GO:0050896	response to stimulus	
	comp55642_c0_seq1			Alanine, aspartate and glutamate metabolism
miR172	comp32954_c0_seq1	GO:0009856	Pollination	Spliceosome
		GO:0044703	multi-organism reproductive process	
		GO:0044706	multi-multicellular organism process	
miR3630	comp701_c0_seq1			
miR390	comp82582_c0_seq1	GO:0015935	small ribosomal subunit	Ribosome
		GO:0022627	cytosolic small ribosomal subunit	
miR396	comp19066_c0_seq1			
	comp89993_c0_seq1			
	comp67855_c0_seq1			
	comp77260_c0_seq1			
	comp54983_c0_seq1			
	comp45923_c0_seq1			
miR482	comp6983_c0_seq1	GO:0006082	organic acid metabolic process	Vitamin B6 metabolism
		GO:0006520	cellular amino acid metabolic process	
		GO:0006950	response to stress	
		GO:0019752	carboxylic acid metabolic process	
		GO:0043436	oxoacid metabolic process	
		GO:0050896	response to stimulus	
miR5083	comp95403_c0_seq1			
miR5538	comp67128_c0_seq1			
miR6300	comp43487_c0_seq1			
miR845	comp32983_c0_seq1			
miR894	comp32058_c0_seq1	GO:0006950	response to stress	
		GO:0044248	cellular catabolic process	

**Fig 7:**
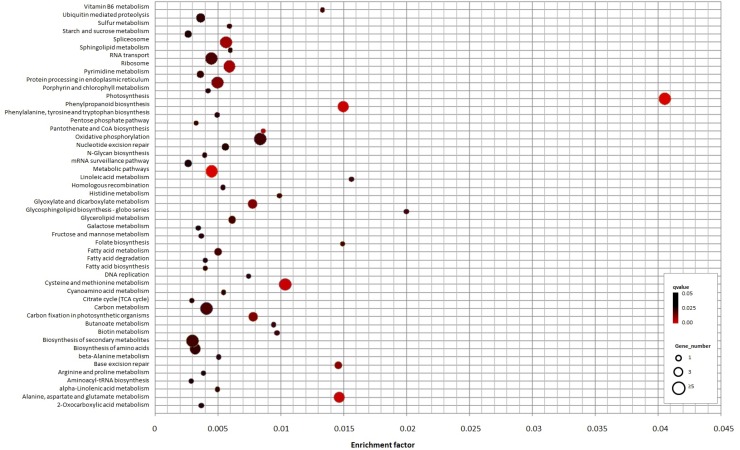
The enriched KEGG pathways of the target genes. The enrichment factor (total number of the specific pathways in the predicted miRNA targets vs. total number of the pathways in the reference olive trascriptome) is on the x-axis. The size of each point represents the number of genes enriched in a particular pathway. A larger enrichment factor value and lower Q-values indicates a greater degree of enrichment.

A total of 58 KEGG pathways were enriched of which 50 were detected at significantly high abundance level (q-value < 0.05) (Fig 7, Table 2, S5 Table). Notably, the most significantly enriched pathways were ʹphotosynthesisʹ (ko00195), ʹmetabolic pathwaysʹ (ko01100) and ʹphenylpropanoid biosynthesisʹ (ko00940). Because these metabolic categories are so broad, however, no pathway maps could be identified in the KEGG database. Nevertheless, several identified pathways have a role in the fruit ripening, like as ʹfatty acid degradation and biosynthesisʹ (Fig 7, Table 2, S5 Table).

Interestingly, most of the annotated transcripts involved in phenylpropanoid regulation are target of miRNAs up-regulated in Cassanese cv (miR156 and miR168), suggesting a key role of this miRNA family in the regulation of colour transition in olive (Table 2).

### Real-time quantification of miRNAs and their target genes

**Fig 8:**
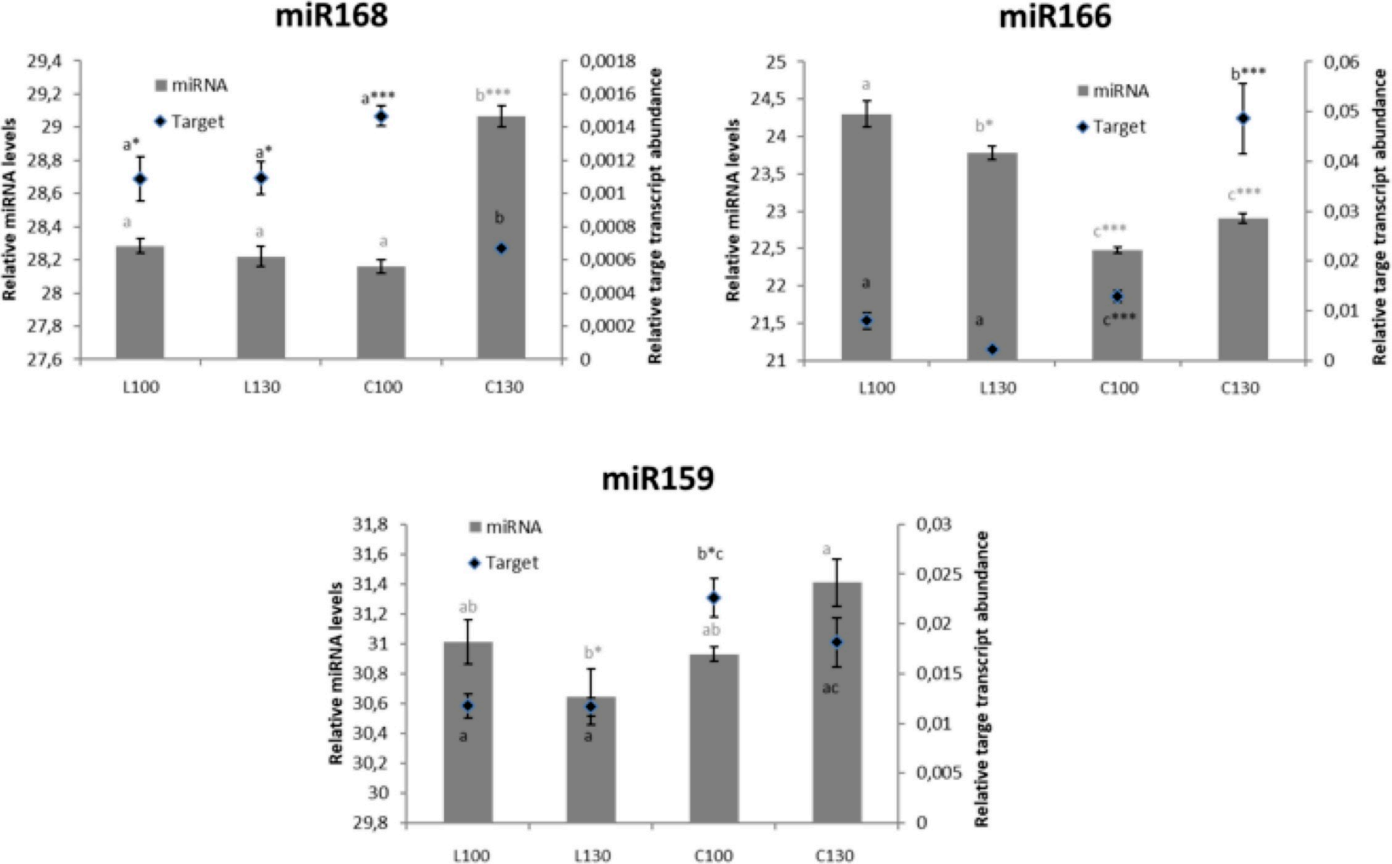
qRT-PCR of selected miRNAs and their target transcripts. The histograms show the relative values of miRNAs while the lines represent the relative abundance of target transcripts. The analyses were performed as triplicates and the error bars indicate the standard error of the mean (s.e.m.). For each value, different letters indicate significant differences among mean values (*p-value ≤ 0.05; ** *p*-value ≤ 0.01; *** *p*-value ≤ 0.001). For all variables with the same letter, the difference between the means is not statistically significant.

In this study, the expression levels of three miRNAs (miR159, miR166 and miR168) and their respective target genes (comp19510, comp97992 and com60984) were additionally analysed by Real-time qRT-PCR. In order to obtain a clear view of the miRNA regulation genotype and ripening-stage specific, we excluded potential environmental effects, as temperature, rainfall and age of plants, by collecting similar samples four years later those obtained for deep sequencing. Moreover, we preferred collect drupes at more late ripening stages to better appreciate differences between two genotypes. Notably, as expected, the results showed that the miRNAs were negatively correlated with the expression level of their targets (Fig 8). MiR168 was highly expressed at 130 DAF in Cassanese coloured cv, while its target gene was down regulated (Fig 8). On the other hand, miR166 was strongly expressed in white-coloured ʹLeucocarpaʹ in both ripening stages, while its target was down regulated (Fig 8). For the last analyzed miRNA (miR159), although less marked than the other analysed miRNAs, we observed a clear negative correlation between miRNA and its target expression levels, with a substantial down regulation of miR159 and up regulation of its target in Cassanese cv and an opposite trend in Leucocarpa cv (Fig 8).

Comparing PCR data with results of the deep sequencing, for two of the studied miRNAs (miR166, miR168) we observed a similar expression pattern during ripening in both genotypes.

## Discussion

Fruit development and ripening is an effect of a fine harmonization of biochemical pathways leading to changes in colour, texture, nutrient accumulation and aroma. Some of these pathways are related to plant secondary metabolism and lead to the production of several compounds such as carotenoids and different phenol classes, including anthocyanins, which may also play an important role in the fruit postharvest performance. Namely, acting as antioxidants, they prevent lipid peroxidation, and maintain membrane integrity to decelerate cell senescence [70]. Accordingly, tomato, fruits enriched in anthocyanins in the epicarp, show less over-ripening and extend their shelf-life [71, 72]. Anthocyanins are also important phytonutrients in a healthy diet, exerting anti-tumour, pro-apoptotic, anti-oxidative, anti-inflammatory and anti-neurodegenerative properties [37, 39, 40, 73, 74].

In the last years many approaches were used to elucidate the molecular aspects of fruit development and ripening in a lot of plant species and recently thanks to easier access to the high-throughput sequencing technology, an enormous amount of genome data and gene expression profiles was produced. Some of these data deal specifically with the identification of several miRNAs which have been found to play key roles in the regulation of diverse biological processes and metabolic pathway [75, 76], including fruit development and ripening [77–79] as well as the biosynthesis and accumulation of secondary metabolites in plants such as flavonoids [32, 33]. In particular, it has been demonstrated that miRNAs interact with the major branches of phenylpropanoid pathway along which flavonoid backbone is synthesized [33].

Concerning olive, although two studies have been carried to identify and characterize miRNAs in *Olea europaea* [21, 69], in both cases no different varieties have been compared in order to highlights possible correlations between fruit pigmentation and ripening process genotype-dependent. Previously, we documented in two olive varieties, ʹLeucocarpaʹ, characterized by a switch-off in skin colour of ripe drupes, and ʹCassaneseʹ used as control plant, significant differences in anthocyanin transcript profiles both ripening and genotype dependent [57]. In this study, four drupe samples taken during 100–130 DAF transition and from Leucocarpa and Cassanese varieties were analysed, aiming to elucidate miRNA putative roles in the biosynthesis and accumulation of secondary metabolisms and particularly of phenylpropanoids.

This resulted in identification and characterization of 130 known conserved miRNAs across 14 families and 492 unique novel miRNAs. As shown in S2 Table and Fig 3, miR166 was most abundant among conserved families. In line with the presence of miR166 family in all analysed plant species and tissues, it was found in all our libraries confirming an its essential role across the plant kingdom with housekeeping functions [79, 80]. However, it was more than 2-fold down regulated in ripe drupes of Leucocarpa cv respect to the three other samples. miR166 was predicted to target three transcripts involved in cell proliferation, catabolic processes resulting in cell wall disassembly (Table 2). Moreover, in a previous study of its significant up regulation was observed in the unripe olive fruits, suggesting a potential inhibition of the fruit maturation by suppression of these target transcripts [21]. Our qRT-PCR assays confirmed this hypothesis showing an opposite trend, in the control variety (Cassanese), between miR166 and its target, up and down regulated in the late ripening stage, respectively (Fig 8). Interestingly, in the white coloured ʹLeucocarpaʹ, miR166 expression was found persistently high during the fruit ripening and the levels of its target transcript were down regulated in both ripening stages (Fig 8). It may be speculated that the induction of this miRNA may inhibit some typical ripening processes, like as fruit veraison, by suppressing its target gene. Illumina and qRT-PCR data are comparable within intravarietal ripening profiles while clear differences resulted within intervarietal comparisons. In fact, if on the one hand miR166 was significantly down regulated during ripening only in Leucocarpa cv on the other hand, the divergences in the intervarietal comparisons were probably due to a genotype-dependent responses.

The miR159, also detected in previous studies in olive [21, 69], was the second most abundant conserved miRNA family found in the olive fruit library. It was ubiquitously expressed in all the libraries but showed an opposite expression pattern between ʹLeucocarpaʹ and ʹCassaneseʹ (S2 Table, Table 2, Fig 3). Although qRT-PCR assays confirmed Illumina data highlighting a slight down regulation of miR159 in ‘Cassanese’ during ripening, the differences were not significant (Fig 8). Also, miRNA168 family, which is known to regulate target genes involved in plant development, signal transduction, metabolism, and defense response was detected in our libraries. Their transcripts were higher in ‘Cassanese’ fruit at 130 DAF than 100DAF while in ‘Leucocarpa’ they were unchanged during ripening transition and similar to those of ‘Cassanese’ 100 DAF. Also, the levels of its target gene were down regulated at the last ripening stages, suggesting a putative role in the biosynthesis and/or accumulation of anthocyanins metabolites.

In conclusion, miRNAs play a role in plant metabolism, development, and defense response. Accordingly, our results showed that miRNAs play a role in olive fruit development tightly related to the production of wealthy secondary metabolites, such as anthocyanins which exert benefits for human health. Interestingly miRNA168a and 159a, provided through diet, was able to pass through the mouse and human gastrointestinal track and decrease the LDL levels in the plasma [81, 82] showing that, exogenous miRNAs can regulate target gene expression and recipient cell function. Therefore, it is tempting to suggest that miRNA could represent potential molecules to promote the production of functional food.

Starting from these evidences, the development of the high-throughput sequencing technology and genome-scale approaches provides a powerful approach to investigate small RNA, including miRNAs and their target genes in organisms from model plant species to non-agricultural crops and agricultural species such as in olive.

## Supporting information

S1 Fig Olive fruit branches of *Olea europaea* (A- Cassanese and B- Leucocarpa cultivars).
(PDF)

S2 Fig Raw reads were pre-processed following pipeline illustrate as a flow diagram. (PDF)

S1 Table Sequence of primers used for qPCR. (PDF)

S2 Table Reads counts of known miRNAs. (PDF)

S3 Table Reads counts of putative novel miRNAs. (PDF)

S4 Table Distribution of ontological categories in fruit ripening of Leucocarpa and Cassanese cultivarvs. (TXT)

S5 Table KEGG pathways in fruit ripening of Leucocarpa and Cassanese cultivarvs. (TXT)
