# Correction: Identification of miRNAs involved in fruit ripening by deep sequencing of *Olea europaea* L. transcriptome

**DOI:** 10.1371/journal.pone.0223354

**Published:** 2019-09-30

**Authors:** Fabrizio Carbone, Leonardo Bruno, Gaetano Perrotta, Maria B. Bitonti, Innocenzo Muzzalupo, Adriana Chiappetta

Affiliation 1 and affiliation 2 are incomplete and incorrectly switched. The complete, correct affiliation 1 is: Research Centre for Olive, Citrus and Tree Fruit—Council for Agricultural Research and Economics, 87036 Rende (CS) IT. The complete, correct affiliation 2 is: Department of Biology, Ecology and Earth Science, University of Calabria, 87036 Arcavacata Rende (CS) IT.

Affiliation 3 is incomplete. The complete affiliation 3 is: Agenzia Nazionale per le Nuove Tecnologie, l’Energia e lo Sviluppo Economico Sostenibile (ENEA), TRISAIA Research Center, S.S. 106 Jonica, 75026 Rotondella (MT) IT.
