# Supplementary figures and images for: Identification of miRNAs involved in fruit ripening by deep sequencing of Olea europaea L. transcriptome
Source: PLoS One. 2019 Aug 22;14(8):e0221460. doi: 10.1371/journal.pone.0221460 (PMC6705801; doi:10.1371/journal.pone.0221460)

1 **S1 Fig.**

2

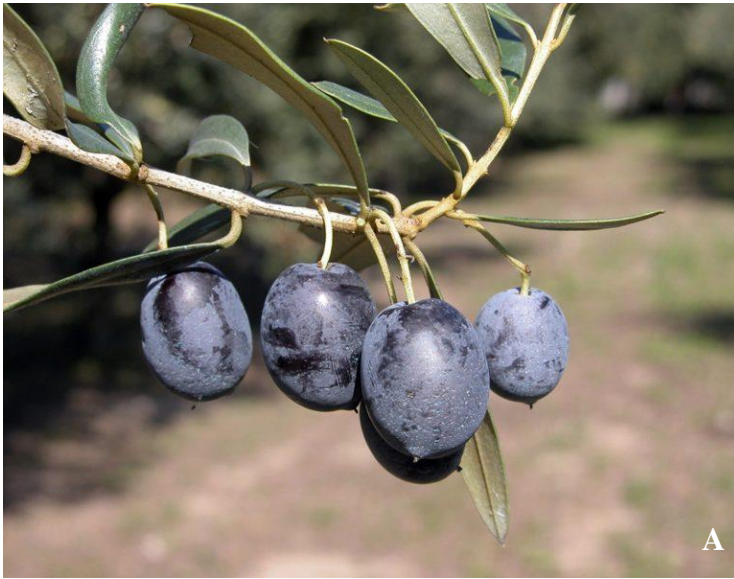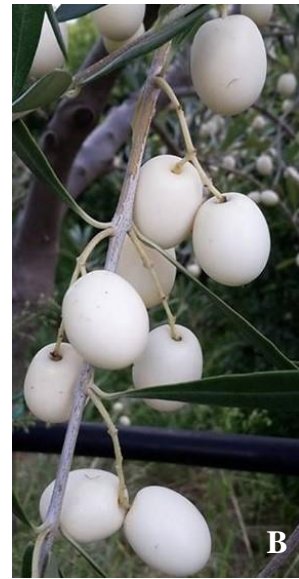

3

Supplement: S1 Fig — Olive fruit branches of Olea europaea (A- Cassanese and B- Leucocarpa cultivars). (PDF) [file pone.0221460.s001.pdf]

# Analysis Pipeline

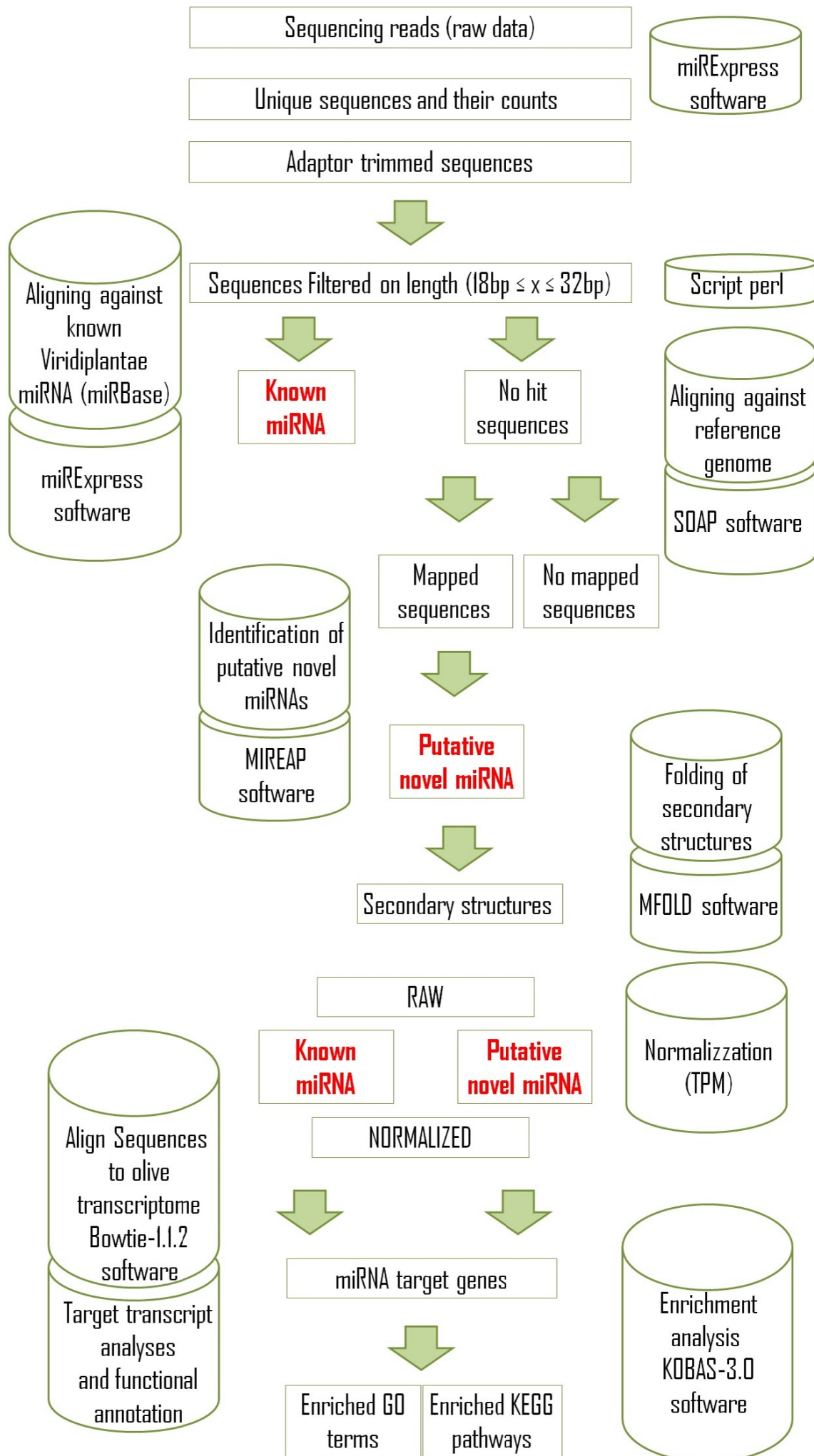

Supplement: S2 Fig — (PDF) [file pone.0221460.s002.pdf]
