# Supplementary material for: Identification of miRNAs involved in fruit ripening by deep sequencing of Olea europaea L. transcriptome
Source: PLoS One. 2019 Aug 22;14(8):e0221460. doi: 10.1371/journal.pone.0221460 (PMC6705801; doi:10.1371/journal.pone.0221460)
Supplement: S1 Table — (PDF) [file pone.0221460.s003.pdf]

S1 Table

A. Stem-loop qRT-PCR

**Oe-miRNAs primers**	**RT Primers (5'->3')**	**Forward Primers(F) (5'->3')**	**Universal Reverse Primer(R) (5'->3')**
Oe-miR159	5’-GTCGTATCCAGTGCAGGGTCCGAGGTATTCGCACTGG-3’	5’-GCGGCGGTATTGGAGTGAAGGGA-3’	5'-GTGCAGGGTCCGAGGT-3'
Oe-miR166		5'-GCGGCGGTCGGACCAGGCTTCAT-3’	
Oe-miR168	5'-GTCGTATCCAGTGCAGGGTCCGAGGTATTCGCACTGGATACGACATTCAC-3'	5'-TTCCTTGATCCCGCCTTGCACCAA-3'	

From Yanik et al., 2013

B. Quantitative Real-time PCR of Predicted Target Genes

**Sequences**	**Target primers**
5’-CTATGATCGCATCCTCCG-3'	comp60984_forw
5’-CTATTGAGCATTAGCAGGTCG-3'	comp60984_rev
5’-CGCGCTATAGGTCAGTATCA-3'	comp19510_rev
5’-GACAAAGTGGGAGTTGGATG-3'	comp19510_forw
5’-CCAATATCATCCCAGTGAGC-3'	comp97992_rev
5’-GAAGGTGATCTCGAGGTGTG-3'	comp97992_forw
