# Supplementary material for: Identification of miRNAs involved in fruit ripening by deep sequencing of Olea europaea L. transcriptome
Source: PLoS One. 2019 Aug 22;14(8):e0221460. doi: 10.1371/journal.pone.0221460 (PMC6705801; doi:10.1371/journal.pone.0221460)
Supplement: S2 Table — (PDF) [file pone.0221460.s004.pdf]

## S2 Table

| | raw data | | | | nomalized data | | | |
|---|---|---|---|---|---|---|---|---|
| **miRNA_Name** | **C100** | **C130** | **L100** | **L130** | **C100** | **C130** | **L100** | **L130** |
| miR156 | 6 | 6 | 6 | 0 | 3 | 1 | 2 | 0 |
| miR156a | 20 | 32 | 20 | 16 | 8 | 5 | 7 | 4 |
| miR156a-5p | 5 | 5 | 5 | 0 | 2 | 1 | 2 | 0 |
| miR156b | 15 | 21 | 15 | 8 | 6 | 3 | 5 | 2 |
| miR156b-5p | 8 | 8 | 8 | 0 | 4 | 1 | 3 | 0 |
| miR156c | 14 | 17 | 14 | 4 | 6 | 3 | 5 | 1 |
| miR156c-5p | 6 | 6 | 6 | 0 | 3 | 1 | 2 | 0 |
| miR156d | 14 | 14 | 14 | 0 | 6 | 2 | 5 | 0 |
| miR156d-5p | 8 | 8 | 8 | 0 | 4 | 1 | 3 | 0 |
| miR156e | 9 | 12 | 9 | 4 | 4 | 2 | 3 | 1 |
| miR156e-5p | 5 | 5 | 5 | 0 | 2 | 1 | 2 | 0 |
| miR156f | 5 | 8 | 5 | 4 | 2 | 1 | 2 | 1 |
| miR156f-5p | 6 | 6 | 6 | 0 | 3 | 1 | 2 | 0 |
| miR156g | 5 | 5 | 5 | 0 | 2 | 1 | 2 | 0 |
| miR156g-5p | 5 | 5 | 5 | 0 | 2 | 1 | 2 | 0 |
| miR156h | 3 | 3 | 3 | 0 | 2 | 1 | 1 | 0 |
| miR156h-5p | 4 | 4 | 4 | 0 | 2 | 1 | 2 | 0 |
| miR156i | 4 | 4 | 4 | 0 | 2 | 1 | 2 | 0 |
| miR156i-5p | 4 | 4 | 4 | 0 | 2 | 1 | 2 | 0 |
| miR156j | 2 | 5 | 2 | 4 | 1 | 1 | 1 | 1 |
| miR156j-5p | 2 | 2 | 2 | 0 | 1 | 0 | 1 | 0 |
| miR156k | 2 | 5 | 2 | 4 | 1 | 1 | 1 | 1 |
| miR156k-5p | 1 | 1 | 1 | 0 | 1 | 0 | 1 | 0 |
| miR156l | 1 | 1 | 1 | 0 | 1 | 0 | 1 | 0 |
| miR156l-5p | 1 | 1 | 1 | 0 | 1 | 0 | 1 | 0 |
| miR156m | 1 | 1 | 1 | 0 | 1 | 0 | 1 | 0 |
| miR156n | 2 | 2 | 2 | 0 | 1 | 0 | 1 | 0 |
| miR156o | 2 | 2 | 2 | 0 | 1 | 0 | 1 | 0 |
| miR156q | 1 | 1 | 1 | 0 | 1 | 0 | 1 | 0 |
| miR156s | 1 | 1 | 1 | 0 | 1 | 0 | 1 | 0 |
| miR156t | 0 | 3 | 0 | 4 | 0 | 1 | 0 | 1 |
| miR156u | 1 | 4 | 1 | 4 | 1 | 1 | 1 | 1 |
| miR156v | 1 | 4 | 1 | 4 | 1 | 1 | 1 | 1 |
| miR156w | 1 | 4 | 1 | 4 | 1 | 1 | 1 | 1 |
| miR156x | 1 | 1 | 1 | 0 | 1 | 0 | 1 | 0 |
| miR156y | 1 | 1 | 1 | 0 | 1 | 0 | 1 | 0 |
| miR159 | 77 | 697 | 324 | 152 | 31 | 97 | 105 | 39 |
| miR159a | 120 | 1102 | 538 | 228 | 49 | 154 | 174 | 59 |
| miR159a-3p | 23 | 213 | 102 | 44 | 10 | 30 | 33 | 12 |
| miR159b | 43 | 402 | 214 | 76 | 18 | 56 | 69 | 20 |
| miR159b-3p | 20 | 194 | 112 | 32 | 8 | 27 | 36 | 8 |
| miR159c | 16 | 130 | 65 | 26 | 7 | 18 | 21 | 7 |
| miR159c-3p | 2 | 0 | 0 | 0 | 1 | 0 | 0 | 0 |
| miR159d | 5 | 48 | 28 | 8 | 2 | 7 | 9 | 2 |
| miR159e-3p | 9 | 82 | 37 | 18 | 4 | 12 | 12 | 5 |
| miR159f | 0 | 3 | 0 | 1 | 0 | 1 | 0 | 1 |
| miR159f-3p | 5 | 48 | 28 | 8 | 2 | 7 | 9 | 2 |
| miR159j-3p | 5 | 48 | 28 | 8 | 2 | 7 | 9 | 2 |
| miR159k-3p | 5 | 48 | 28 | 8 | 2 | 7 | 9 | 2 |
| miR166 | 438 | 1282 | 524 | 259 | 176 | 179 | 169 | 67 |
| miR166a | 6170 | 17348 | 6944 | 3422 | 2480 | 2419 | 2236 | 877 |
| miR166a-3p | 1740 | 5043 | 2074 | 1009 | 700 | 703 | 668 | 259 |

| | | | | | | | | |
|---|---|---|---|---|---|---|---|---|
| miR166b | 5289 | 15167 | 6139 | 3008 | 2126 | 2115 | 1977 | 771 |
| miR166b-3p | 1326 | 3911 | 1582 | 790 | 533 | 545 | 510 | 203 |
| miR166c | 4382 | 12493 | 5052 | 2469 | 1762 | 1742 | 1627 | 633 |
| miR166c-3p | 1547 | 4401 | 1761 | 874 | 622 | 614 | 567 | 224 |
| miR166d | 3737 | 10736 | 4359 | 2139 | 1502 | 1497 | 1404 | 549 |
| miR166d-3p | 1326 | 3906 | 1578 | 786 | 533 | 545 | 508 | 202 |
| miR166e | 3275 | 9246 | 3739 | 1821 | 1317 | 1289 | 1204 | 467 |
| miR166e-3p | 1290 | 3691 | 1536 | 735 | 519 | 515 | 495 | 189 |
| miR166f | 3093 | 8684 | 3443 | 1701 | 1244 | 1211 | 1109 | 436 |
| miR166f-3p | 213 | 605 | 255 | 119 | 86 | 85 | 82 | 31 |
| miR166g | 1970 | 5731 | 2347 | 1147 | 792 | 799 | 756 | 294 |
| miR166g-3p | 904 | 2586 | 997 | 511 | 364 | 361 | 321 | 131 |
| miR166h | 1757 | 5126 | 2092 | 1026 | 707 | 715 | 674 | 263 |
| miR166h-3p | 671 | 1846 | 719 | 354 | 270 | 258 | 232 | 91 |
| miR166i | 1082 | 3088 | 1281 | 614 | 435 | 431 | 413 | 158 |
| miR166i-3p | 663 | 1953 | 789 | 393 | 267 | 272 | 254 | 101 |
| miR166j | 883 | 2382 | 960 | 462 | 355 | 332 | 309 | 119 |
| miR166j-3p | 230 | 686 | 273 | 136 | 93 | 96 | 88 | 35 |
| miR166k | 917 | 2477 | 927 | 474 | 369 | 346 | 299 | 122 |
| miR166l | 222 | 610 | 256 | 121 | 90 | 85 | 83 | 31 |
| miR166l-3p | 241 | 634 | 208 | 118 | 97 | 89 | 67 | 31 |
| miR166m | 239 | 696 | 277 | 140 | 96 | 97 | 89 | 36 |
| miR166m-3p | 228 | 555 | 191 | 99 | 92 | 78 | 62 | 26 |
| miR166n | 240 | 764 | 291 | 159 | 97 | 107 | 94 | 41 |
| miR166o | 240 | 764 | 291 | 159 | 97 | 107 | 94 | 41 |
| miR166p | 213 | 600 | 251 | 117 | 86 | 84 | 81 | 30 |
| miR166q | 240 | 764 | 291 | 159 | 97 | 107 | 94 | 41 |
| miR166r | 6 | 11 | 4 | 0 | 3 | 2 | 2 | 0 |
| miR166s | 225 | 677 | 269 | 138 | 91 | 95 | 87 | 36 |
| miR166t | 225 | 677 | 269 | 138 | 91 | 95 | 87 | 36 |
| miR166u | 225 | 676 | 269 | 138 | 91 | 94 | 87 | 36 |
| miR168 | 18 | 108 | 135 | 81 | 8 | 15 | 44 | 21 |
| miR168a | 12 | 72 | 90 | 59 | 5 | 10 | 29 | 15 |
| miR168a-3p | 6 | 22 | 3 | 10 | 3 | 3 | 1 | 3 |
| miR168a-5p | 8 | 48 | 60 | 41 | 4 | 7 | 20 | 11 |
| miR168b | 8 | 48 | 60 | 41 | 4 | 7 | 20 | 11 |
| miR168b-3p | 3 | 15 | 0 | 9 | 2 | 2 | 0 | 3 |
| miR168b-5p | 6 | 36 | 45 | 37 | 3 | 5 | 15 | 10 |
| miR168c | 0 | 0 | 0 | 5 | 0 | 0 | 0 | 2 |
| miR168c-3p | 2 | 10 | 0 | 6 | 1 | 2 | 0 | 2 |
| miR168c-5p | 2 | 12 | 15 | 14 | 1 | 2 | 5 | 4 |
| miR168d | 2 | 12 | 15 | 9 | 1 | 2 | 5 | 3 |
| miR168e | 2 | 12 | 15 | 9 | 1 | 2 | 5 | 3 |
| miR172d | 0 | 3 | 0 | 0 | 0 | 1 | 0 | 0 |
| miR3630-3p | 1 | 0 | 0 | 0 | 1 | 0 | 0 | 0 |
| miR390 | 0 | 28 | 0 | 12 | 0 | 4 | 0 | 3 |
| miR390-5p | 0 | 14 | 0 | 6 | 0 | 2 | 0 | 2 |
| miR390a | 0 | 52 | 0 | 22 | 0 | 7 | 0 | 6 |
| miR390a-5p | 0 | 30 | 0 | 12 | 0 | 4 | 0 | 3 |
| miR390b | 0 | 56 | 0 | 24 | 0 | 8 | 0 | 6 |
| miR390b-5p | 0 | 28 | 0 | 10 | 0 | 4 | 0 | 3 |
| miR390c | 0 | 34 | 0 | 14 | 0 | 5 | 0 | 4 |
| miR390d | 0 | 16 | 0 | 6 | 0 | 2 | 0 | 2 |
| miR390d-5p | 0 | 4 | 0 | 2 | 0 | 1 | 0 | 1 |
| miR390e | 0 | 10 | 0 | 4 | 0 | 2 | 0 | 1 |
| miR390f | 0 | 10 | 0 | 4 | 0 | 2 | 0 | 1 |

| | | | | | | | | |
|---|---|---|---|---|---|---|---|---|
| miR390g | 0 | 6 | 0 | 2 | 0 | 1 | 0 | 1 |
| miR396 | 0 | 0 | 0 | 6 | 0 | 0 | 0 | 2 |
| miR396-5p | 0 | 0 | 0 | 6 | 0 | 0 | 0 | 2 |
| miR396a | 0 | 0 | 0 | 4 | 0 | 0 | 0 | 1 |
| miR396a-3p | 0 | 0 | 0 | 1 | 0 | 0 | 0 | 1 |
| miR396a-5p | 0 | 0 | 0 | 4 | 0 | 0 | 0 | 1 |
| miR396b | 0 | 0 | 0 | 18 | 0 | 0 | 0 | 5 |
| miR396b-5p | 0 | 0 | 0 | 6 | 0 | 0 | 0 | 2 |
| miR396c | 0 | 0 | 0 | 19 | 0 | 0 | 0 | 5 |
| miR396c-5p | 0 | 0 | 0 | 4 | 0 | 0 | 0 | 1 |
| miR396d | 0 | 0 | 0 | 8 | 0 | 0 | 0 | 2 |
| miR396e | 0 | 0 | 0 | 10 | 0 | 0 | 0 | 3 |
| miR396e-5p | 0 | 0 | 0 | 6 | 0 | 0 | 0 | 2 |
| miR396f | 0 | 0 | 0 | 2 | 0 | 0 | 0 | 1 |
| miR396f-5p | 0 | 0 | 0 | 2 | 0 | 0 | 0 | 1 |
| miR396k-5p | 0 | 0 | 0 | 2 | 0 | 0 | 0 | 1 |
| miR482c-3p | 2 | 0 | 0 | 0 | 1 | 0 | 0 | 0 |
| miR5083 | 0 | 0 | 0 | 1 | 0 | 0 | 0 | 1 |
| miR5538 | 0 | 0 | 0 | 2 | 0 | 0 | 0 | 1 |
| miR6300 | 1 | 3 | 0 | 1 | 1 | 1 | 0 | 1 |
| miR845c | 1 | 0 | 0 | 0 | 1 | 0 | 0 | 0 |
| miR894 | 1 | 20 | 6 | 0 | 1 | 3 | 2 | 0 |

1
