# Supplementary material for: Identification of miRNAs involved in fruit ripening by deep sequencing of Olea europaea L. transcriptome
Source: PLoS One. 2019 Aug 22;14(8):e0221460. doi: 10.1371/journal.pone.0221460 (PMC6705801; doi:10.1371/journal.pone.0221460)
Supplement: S3 Table — (PDF) [file pone.0221460.s005.pdf]

## S3 Table

| Novel ID | Location | Strand | Sequence of 3’ | Sequence of 5’ | raw data | | | | nomalized data | | | |
|---|---|---|---|---|---|---|---|---|---|---|---|---|
| | | | | | C100 | C130 | L100 | L130 | C100 | C130 | L100 | L130 |
| Oe_mir_1 | FKYM01055610.1_2875:2902 | - | | CAGATGCAATCTGCACCACCCCAGGGTT | 6 | 0 | 0 | 4 | 3 | 0 | 0 | 1 |
| Oe_mir_2 | FKYM01055214.1_1219:1247 | + | GAGAGGGATGGGGTTTCTCTCGCTTTTGG | | 14 | 0 | 0 | 0 | 6 | 0 | 0 | 0 |
| Oe_mir_3 | FKYM01054197.1_28858:28886 | + | | TTGGGCTCCGGGAGGGGGAGATGTAAGAA | 36 | 0 | 0 | 0 | 15 | 0 | 0 | 0 |
| Oe_mir_4 | FKYM01050891.1_28768:28796 | - | | TGTGCTTGGGAGTCCCTAATGATTAAATA | 4 | 0 | 0 | 0 | 2 | 0 | 0 | 0 |
| Oe_mir_5 | FKYM01050338.1_34826:34853 | + | | GCTTTTGTTTACCGAGGGTTCGAATCCC | 3 | 3 | 0 | 0 | 2 | 1 | 0 | 0 |
| Oe_mir_6 | FKYM01046616.1_5824:5851 | - | | TGATCTGCCGAAGCCGTGGGATGAAAAA | 22 | 0 | 0 | 0 | 9 | 0 | 0 | 0 |
| Oe_mir_7 | FKYM01046010.1_24248:24276 | - | | CTTGAAGAGTTATAGAATGTTCTGAGGCC | 5 | 0 | 0 | 0 | 2 | 0 | 0 | 0 |
| Oe_mir_8 | FKYM01044337.1_45579:45608 | + | TTCTGATGATAAAAGGCGAATCTGAGCCTC | | 93 | 86 | 0 | 15 | 38 | 12 | 0 | 4 |
| Oe_mir_9 | FKYM01043744.1_6792:6820 | - | CAATGATCAGTAGCTGGTTCGAGAGGATG | | 3 | 0 | 0 | 0 | 2 | 0 | 0 | 0 |
| Oe_mir_10 | FKYM01042526.1_21612:21641 | - | CTGTAGCTCAGTTGGTAGAGCACCTCGAAA | | 23 | 0 | 0 | 0 | 10 | 0 | 0 | 0 |
| Oe_mir_11 | FKYM01041785.1_40379:40407 | - | | AGTTTAGGTGAGGTATATTTCTGAGCCAG | 4 | 0 | 0 | 0 | 2 | 0 | 0 | 0 |
| Oe_mir_12 | FKYM01039660.1_42011:42040 | - | AGTAGAGTTGAATAGCGGGAAGATCCAATA | | 3 | 0 | 0 | 0 | 2 | 0 | 0 | 0 |
| Oe_mir_13 | FKYM01038462.1_2970:2999 | + | CCCTTAGATGTTCTGGGCCGCACGCGCGCT | | 5 | 0 | 0 | 0 | 2 | 0 | 0 | 0 |
| Oe_mir_14 | FKYM01036130.1_5095:5123 | + | TATTTGTACAGATATGGTAGAGGGGCACA | | 27 | 0 | 0 | 0 | 11 | 0 | 0 | 0 |
| Oe_mir_15 | FKYM01035635.1_17875:17902 | + | TTGGCATTGAGTTCATGGACCTGTACAG | | 4 | 0 | 0 | 0 | 2 | 0 | 0 | 0 |
| Oe_mir_16 | FKYM01035436.1_188744:188771 | + | | GTGGCCAAGTGGTAAGGCAACGGGTTTT | 35 | 0 | 0 | 0 | 14 | 0 | 0 | 0 |
| Oe_mir_17 | FKYM01033727.1_28864:28894 | - | CGTAGCCGCTCATGGTTATTTTGGCCGATTA | | 6 | 0 | 0 | 0 | 3 | 0 | 0 | 0 |
| Oe_mir_18 | FKYM01033545.1_10576:10604 | + | ACCCCCATGTGAGAGGCCCTGTACAGACC | | 4 | 0 | 0 | 0 | 2 | 0 | 0 | 0 |
| Oe_mir_19 | FKYM01033405.1_36717:36744 | - | | GAGAGAAGAGCTGGAGCTGAAAATGAGT | 3 | 3 | 0 | 0 | 2 | 1 | 0 | 0 |
| Oe_mir_20 | FKYM01031732.1_27775:27803 | - | | TAGACCTTGTTGTTGTCAGAATTCTTAAT | 3 | 0 | 0 | 0 | 2 | 0 | 0 | 0 |
| Oe_mir_21 | FKYM01031203.1_21800:21828 | + | TCACTGGTCTAGCTCCATGGCACTAAAAA | | 7 | 0 | 0 | 6 | 3 | 0 | 0 | 2 |
| Oe_mir_22 | FKYM01031021.1_16233:16261 | + | | GGACCAGACAGGCGCAGGTAGCTTGACCG | 5 | 0 | 0 | 0 | 2 | 0 | 0 | 0 |
| Oe_mir_23 | FKYM01030694.1_9539:9567 | - | CGAGGGCTACTATAGTTGATATGTGGAGC | | 7 | 26 | 0 | 0 | 3 | 4 | 0 | 0 |
| Oe_mir_24 | FKYM01030229.1_16611:16641 | - | CCGCGCGATGTCGACGTCGAAGAGGAATGCT | | 51 | 0 | 0 | 0 | 21 | 0 | 0 | 0 |
| Oe_mir_25 | FKYM01029890.1_12726:12756 | - | GATGTCGATCCTGAGCGGTTACCTGAGCAAC | | 23 | 0 | 0 | 15 | 10 | 0 | 0 | 4 |
| Oe_mir_26 | FKYM01025752.1_3968:3999 | + | CTTGTTGATTGAGAATCGTATCTGTGGCTACT | | 11 | 0 | 0 | 0 | 5 | 0 | 0 | 0 |
| Oe_mir_27 | FKYM01024763.1_626:656 | - | GGGGATGCCGAAGGCAGGGCTAGTGACTGGA | | 12 | 0 | 0 | 0 | 5 | 0 | 0 | 0 |
| Oe_mir_28 | FKYM01022360.1_5031:5060 | - | CTTGGGCGAGAGTAGTACTAGGATGGGTGA | | 78 | 0 | 0 | 0 | 32 | 0 | 0 | 0 |
| Oe_mir_29 | FKYM01022360.1_11953:11982 | - | CCCCCTGGGAAGTCCTCGTGTTGCACCCCT | | 19 | 150 | 0 | 0 | 8 | 21 | 0 | 0 |

| | | | | | | | | | | | | |
|---|---|---|---|---|---|---|---|---|---|---|---|---|
| Oe_mir_30 | FKYM01022360.1_16605:16636 | - | | TAGTACTAGGATGGGTGACCCCCTGGGAAGTT | 1 | 0 | 0 | 0 | 1 | 0 | 0 | 0 |
| Oe_mir_31 | FKYM01022360.1_16257:16286 | - | CCCCCTGGGAAGTCCTCGTGTTGTACCCCT | | 8 | 0 | 0 | 0 | 4 | 0 | 0 | 0 |
| Oe_mir_32 | FKYM01022358.1_15344:15372 | - | | CCCCTGTGAAGTCCTCGTGTTGCACCCCT | 9 | 131 | 34 | 29 | 4 | 18 | 11 | 8 |
| Oe_mir_33 | FKYM01022358.1_20325:20353 | - | | CCCCTGGGAAGTTCTCGTGTTGCACCCCT | 5 | 48 | 0 | 0 | 2 | 7 | 0 | 0 |
| Oe_mir_34 | FKYM01022358.1_31057:31084 | - | AGTAGTACTAGGATGGGTGACCCTCTGG | | 3 | 0 | 0 | 4 | 2 | 0 | 0 | 1 |
| Oe_mir_35 | FKYM01020931.1_11846:11876 | + | | AGCCATTCGGCGGTGAATTCGTTCCCGGGCC | 5 | 0 | 0 | 0 | 2 | 0 | 0 | 0 |
| Oe_mir_36 | FKYM01014275.1_38979:39010 | + | | ATTTGGTCTAGTGGTATGATTCTCGCTTTGGG | 4 | 0 | 0 | 0 | 2 | 0 | 0 | 0 |
| Oe_mir_37 | FKYM01014067.1_11568:11599 | + | AGGTGTAGCAATAATGAACGCTGGTGATGTTG | | 12 | 20 | 0 | 0 | 5 | 3 | 0 | 0 |
| Oe_mir_38 | FKYM01007076.1_17288:17316 | + | GTGAGGCTGGGCGACCTGATGAGGTGGCA | | 12 | 0 | 0 | 6 | 5 | 0 | 0 | 2 |
| Oe_mir_39 | FKYM01006094.1_22386:22416 | + | AGTGGTAAGAATTCCACGTTGTGGCCGTGGA | | 24 | 0 | 0 | 0 | 10 | 0 | 0 | 0 |
| Oe_mir_40 | FKYM01002722.1_3841:3872 | + | | GCCTCGTGGACTCCGTTGTCTGAATACTGTAT | 3 | 0 | 0 | 0 | 2 | 0 | 0 | 0 |
| Oe_mir_41 | FKYM01002369.1_752:781 | - | TTATTGTCTATCGTCGGCCTCTATGGTAGA | | 18 | 0 | 0 | 0 | 8 | 0 | 0 | 0 |
| Oe_mir_42 | FKYM01000663.1_3464:3493 | - | TACTTCTTCCTAGCTGCTTGGCCTGTAGTA | | 117 | 0 | 0 | 0 | 47 | 0 | 0 | 0 |
| Oe_mir_43 | FKYM01052587.1_72930:72958 | + | | TGCATCGCGTGACAAATCTATTAAGCTTT | 5 | 24 | 0 | 0 | 2 | 3 | 0 | 0 |
| Oe_mir_44 | FKYM01051081.1_21848:21875 | - | AGCGACATTATTGTGGAATGTTAGACAC | | 12 | 0 | 0 | 0 | 5 | 0 | 0 | 0 |
| Oe_mir_45 | FKYM01046624.1_613:643 | - | | CAGACCAAGAACTACGAGATCACCCCTTTCA | 16 | 0 | 0 | 0 | 7 | 0 | 0 | 0 |
| Oe_mir_46 | FKYM01044697.1_17668:17695 | - | TCTGATTGTGTTTCTTTTTTTGGTGCAG | | 7 | 45 | 14 | 6 | 3 | 6 | 5 | 2 |
| Oe_mir_47 | FKYM01044337.1_45577:45608 | + | TCTTCTGATGATAAAAGGCGAATCTGAGCCTC | | 45 | 0 | 0 | 0 | 18 | 0 | 0 | 0 |
| Oe_mir_48 | FKYM01043238.1_8907:8935 | + | GTAGCTCATATGGTAGGTCGTTAGTTTTG | | 4 | 0 | 0 | 0 | 2 | 0 | 0 | 0 |
| Oe_mir_49 | FKYM01042526.1_21613:21641 | - | CTGTAGCTCAGTTGGTAGAGCACCTCGAA | | 38 | 62 | 64 | 44 | 16 | 9 | 21 | 12 |
| Oe_mir_50 | FKYM01040697.1_6755:6784 | - | | ACTTCGACCAGCGAACTCGAACAGAATTCC | 3 | 0 | 0 | 0 | 2 | 0 | 0 | 0 |
| Oe_mir_51 | FKYM01038316.1_13055:13084 | - | CTGGAGTTATAACATTTGAATTTCTCTGAC | | 3 | 0 | 0 | 0 | 2 | 0 | 0 | 0 |
| Oe_mir_52 | FKYM01036652.1_25672:25699 | + | | CTTGGTGGTGAAATGGTAGACACGCGAG | 6 | 0 | 0 | 18 | 3 | 0 | 0 | 5 |
| Oe_mir_53 | FKYM01027114.1_15577:15604 | - | AATACTGCTAGACAGGAACATAAGTATG | | 3 | 0 | 0 | 0 | 2 | 0 | 0 | 0 |
| Oe_mir_54 | FKYM01025620.1_64968:64996 | - | | TGATATCGTTGTTGTTGGGTTGCAGGAAG | 3 | 0 | 4 | 0 | 2 | 0 | 2 | 0 |
| Oe_mir_55 | FKYM01024763.1_629:656 | - | GGGGATGCCGAAGGCAGGGCTAGTGACT | | 5 | 0 | 0 | 0 | 2 | 0 | 0 | 0 |
| Oe_mir_56 | FKYM01024477.1_73148:73178 | - | CTGGATGATTTCGGACCAGGCTTCATTCCCC | | 3 | 0 | 0 | 0 | 2 | 0 | 0 | 0 |
| Oe_mir_57 | FKYM01022360.1_11952:11982 | - | | CCCCCTGGGAAGTCCTCGTGTTGCACCCCTT | 66 | 0 | 0 | 0 | 27 | 0 | 0 | 0 |
| Oe_mir_58 | FKYM01022360.1_16257:16285 | - | CCCCTGGGAAGTCCTCGTGTTGTACCCCT | | 15 | 0 | 56 | 21 | 6 | 0 | 18 | 6 |
| Oe_mir_59 | FKYM01022360.1_21160:21189 | - | | TGAGAGTAGTACTAGGATGGGTGACCCCCT | 83 | 0 | 0 | 0 | 34 | 0 | 0 | 0 |
| Oe_mir_60 | FKYM01022358.1_10676:10706 | - | | CCCCTGGGAAGTCCTCGTGTTGCCCCCCTAT | 6 | 0 | 0 | 0 | 3 | 0 | 0 | 0 |
| Oe_mir_61 | FKYM01018308.1_24988:25016 | + | TTCAGCAACGGATTTCTTGGCTCTCGCAT | | 3 | 0 | 0 | 0 | 2 | 0 | 0 | 0 |

| | | | | | | | | | | | | |
|---|---|---|---|---|---|---|---|---|---|---|---|---|
| Oe_mir_62 | FKYM01018276.1_6486:6513 | + | | CCGAGTTGGCGGCTGAGTTGGATTCTAA | 3 | 0 | 0 | 0 | 2 | 0 | 0 | 0 |
| Oe_mir_63 | FKYM01010170.1_31901:31928 | - | CAGTTATAATCCAATTTCTCTGATGGTT | | 18 | 0 | 40 | 0 | 8 | 0 | 13 | 0 |
| Oe_mir_64 | FKYM01002444.1_19730:19757 | + | | GTTGTCTGGCTCGAGAACTCTTTTGTTT | 1 | 0 | 0 | 0 | 1 | 0 | 0 | 0 |
| Oe_mir_65 | FKYM01002444.1_19792:19819 | + | GCGTTGTCGGACCAGGCTTCATTCCCCC | | 171 | 421 | 185 | 95 | 69 | 59 | 60 | 25 |
| Oe_mir_66 | FKYM01001755.1_27381:27411 | + | | GAATTGATGAGCCGATGCGGTCTGAGGTCCA | 3 | 0 | 0 | 0 | 2 | 0 | 0 | 0 |
| Oe_mir_67 | FKYM01001671.1_2100:2131 | + | | GTGCAAAGGTTTCCTCGGGCCGGACGGAGATT | 674 | 0 | 0 | 0 | 271 | 0 | 0 | 0 |
| Oe_mir_68 | FKYM01000663.1_3463:3493 | - | TACTTCTTCCTAGCTGCTTGGCCTGTAGTAG | | 15 | 0 | 0 | 0 | 6 | 0 | 0 | 0 |
| Oe_mir_69 | FKYM01057934.1_3444:3472 | + | | AGTGGTGTTTCCAGTGGCGGACGGGTGAG | 11 | 0 | 0 | 0 | 5 | 0 | 0 | 0 |
| Oe_mir_70 | FKYM01045039.1_60625:60654 | - | | CTACTTAACTCAGTGGTTAGAGTATTGCTT | 4 | 0 | 6 | 0 | 2 | 0 | 2 | 0 |
| Oe_mir_71 | FKYM01043238.1_8907:8934 | + | GTAGCTCATATGGTAGGTCGTTAGTTTT | | 10 | 0 | 0 | 0 | 4 | 0 | 0 | 0 |
| Oe_mir_72 | FKYM01030230.1_110:140 | - | CGACGTCGCGAGAAGTCCACTGAACCTTATC | | 158 | 0 | 0 | 0 | 64 | 0 | 0 | 0 |
| Oe_mir_73 | FKYM01022360.1_15591:15619 | - | | CCCCTGGGAAGTCCTTGTGTTGCACCCCT | 3 | 0 | 0 | 0 | 2 | 0 | 0 | 0 |
| Oe_mir_74 | FKYM01020931.1_11849:11879 | + | | CATTCGGCGGTGAATTCGTTCCCGGGCCTTG | 4 | 0 | 0 | 0 | 2 | 0 | 0 | 0 |
| Oe_mir_75 | FKYM01056150.1_3689:3717 | + | AAAGCTGGGTCATGGACGTCACAATGTTT | | 3 | 0 | 0 | 0 | 2 | 0 | 0 | 0 |
| Oe_mir_76 | FKYM01041963.1_1877:1905 | + | | AATTCAACCTAGTACGAGAGGAACCGTTG | 1 | 0 | 0 | 0 | 1 | 0 | 0 | 0 |
| Oe_mir_77 | FKYM01041963.1_1918:1948 | + | GGTCATCGCGCTTGGTTGAAAAGCCAGTGGC | | 3 | 0 | 0 | 0 | 2 | 0 | 0 | 0 |
| Oe_mir_78 | FKYM01041785.1_40380:40407 | - | | AGTTTAGGTGAGGTATATTTCTGAGCCA | 3 | 18 | 0 | 5 | 2 | 3 | 0 | 2 |
| Oe_mir_79 | FKYM01030229.1_6373:6403 | - | | CGGGACATGCTCGGGCTCGGCTCGTCGCTTG | 1 | 0 | 0 | 0 | 1 | 0 | 0 | 0 |
| Oe_mir_80 | FKYM01030229.1_5912:5941 | - | TGTGAACGACGTTCTTCCTCACCCCGGACC | | 3 | 0 | 0 | 0 | 2 | 0 | 0 | 0 |
| Oe_mir_81 | FKYM01022358.1_16017:16045 | - | CCCCCTGGGAAGTCCTCGTGTTGCACCAC | | 3 | 0 | 0 | 0 | 2 | 0 | 0 | 0 |
| Oe_mir_82 | FKYM01022358.1_37524:37552 | - | CCCTGGGAAGTCCTCGTGTTGCACCCCTT | | 37 | 0 | 0 | 0 | 15 | 0 | 0 | 0 |
| Oe_mir_83 | FKYM01011318.1_22461:22490 | - | CCTACATCGTATTGGACGTAGTGGGCGATT | | 3 | 0 | 0 | 0 | 2 | 0 | 0 | 0 |
| Oe_mir_84 | FKYM01010646.1_44282:44313 | - | TGTGAAGGCATCGGACCAGGCTTCATTCCTCT | | 4 | 0 | 0 | 0 | 2 | 0 | 0 | 0 |
| Oe_mir_85 | FKYM01000663.1_3464:3491 | - | CTTCTTCCTAGCTGCTTGGCCTGTAGTA | | 32 | 0 | 0 | 0 | 13 | 0 | 0 | 0 |
| Oe_mir_86 | FKYM01053456.1_604:634 | + | | CTAGTACGAGAGGACCGGGAAGGACGCACCT | 4 | 0 | 0 | 0 | 2 | 0 | 0 | 0 |
| Oe_mir_87 | FKYM01045973.1_27091:27119 | - | ATGACTTGAGAGGTGTAGGATAAGTGGGA | | 4 | 0 | 0 | 0 | 2 | 0 | 0 | 0 |
| Oe_mir_88 | FKYM01039660.1_42012:42040 | - | AGTAGAGTTGAATAGCGGGAAGATCCAAT | | 4 | 0 | 0 | 0 | 2 | 0 | 0 | 0 |
| Oe_mir_89 | FKYM01030048.1_65397:65424 | - | | CAAGACCGTCGAATTTTGATACCGTTGG | 17 | 0 | 0 | 0 | 7 | 0 | 0 | 0 |
| Oe_mir_90 | FKYM01022360.1_4010:4038 | - | | CCCCTGGGATGTCCTCGTGTTGCACCCCT | 3 | 0 | 0 | 0 | 2 | 0 | 0 | 0 |
| Oe_mir_91 | FKYM01020701.1_5499:5526 | + | | AAATGATGAGTTTGGCACCCTCTGAGTA | 3 | 10 | 4 | 12 | 2 | 2 | 2 | 3 |
| Oe_mir_92 | FKYM01010646.1_44284:44313 | - | TGTGAAGGCATCGGACCAGGCTTCATTCCT | | 6 | 0 | 0 | 0 | 3 | 0 | 0 | 0 |
| Oe_mir_93 | FKYM01002117.1_2497:2526 | + | | GGTGAAGTGTTCGGATCGCGGCGACGTGGG | 6 | 0 | 0 | 0 | 3 | 0 | 0 | 0 |

| | | | | | | | | | | | | |
|---|---|---|---|---|---|---|---|---|---|---|---|---|
| Oe_mir_94 | FKYM01057936.1_3228:3256 | - | | CTCCATCTTCTCTTTTAATCCATTCTTCC | 0 | 3 | 0 | 0 | 0 | 1 | 0 | 0 |
| Oe_mir_95 | FKYM01057852.1_1548:1577 | - | | CCAGTGTTAACTCCAGGAATCGAACTCTCC | 0 | 3 | 0 | 0 | 0 | 1 | 0 | 0 |
| Oe_mir_96 | FKYM01057359.1_22840:22867 | + | | GGTGGAAATCGAGGTGGGGAGGTAATGG | 0 | 3 | 0 | 0 | 0 | 1 | 0 | 0 |
| Oe_mir_97 | FKYM01057189.1_7878:7906 | - | | CTCCTCGGGCAGCCGATCGTAAAGTATTA | 0 | 3 | 0 | 0 | 0 | 1 | 0 | 0 |
| Oe_mir_98 | FKYM01057147.1_46232:46259 | + | | TTTCAGAGCTTGGGGTTCTTAGTTGGAG | 0 | 3 | 0 | 0 | 0 | 1 | 0 | 0 |
| Oe_mir_99 | FKYM01057017.1_21057:21086 | + | TGATGTCAAAGATTGTGGATGGAATTCCTG | | 0 | 3 | 0 | 0 | 0 | 1 | 0 | 0 |
| Oe_mir_100 | FKYM01056678.1_69919:69947 | + | GAGACCTCAGCCTACTAACTAGCTATGCA | | 0 | 4 | 0 | 0 | 0 | 1 | 0 | 0 |
| Oe_mir_101 | FKYM01056600.1_2135:2164 | + | TGATTTGAAGATATGGCAGAGGAAAACGTT | | 0 | 7 | 0 | 0 | 0 | 1 | 0 | 0 |
| Oe_mir_102 | FKYM01056569.1_38446:38475 | - | AATTGTCGCTCCGATTCTTTTTTTTTTTTT | | 0 | 3 | 0 | 0 | 0 | 1 | 0 | 0 |
| Oe_mir_103 | FKYM01056501.1_3277:3305 | - | | CTTGACTGTGGTCCCTGTTGTGCTGTAAG | 0 | 3 | 0 | 0 | 0 | 1 | 0 | 0 |
| Oe_mir_104 | FKYM01055520.1_16794:16823 | + | TAGTGTAGACAAGGTGCTTCGGCAGGACGG | | 0 | 4 | 0 | 0 | 0 | 1 | 0 | 0 |
| Oe_mir_105 | FKYM01055512.1_46275:46302 | - | | TCTGGAGTTATAGTGAAAGGTTCACCTG | 0 | 3 | 0 | 0 | 0 | 1 | 0 | 0 |
| Oe_mir_106 | FKYM01055253.1_22607:22636 | + | | CGGGTTCGAATCTTTGGTTGGCACTTCCTT | 0 | 4 | 0 | 0 | 0 | 1 | 0 | 0 |
| Oe_mir_107 | FKYM01054560.1_1592:1619 | - | CTCGTGACCAAAGGCCTCCGCGTTCACC | | 0 | 4 | 0 | 0 | 0 | 1 | 0 | 0 |
| Oe_mir_108 | FKYM01053416.1_1717:1746 | - | | CATTTGGTCTAGTGGTATGATTCTCGCTTT | 0 | 17 | 0 | 0 | 0 | 3 | 0 | 0 |
| Oe_mir_109 | FKYM01053369.1_3095:3126 | - | CCTGCCACACCTCCACGCGCACTCTCTGGCCT | | 0 | 3 | 0 | 0 | 0 | 1 | 0 | 0 |
| Oe_mir_110 | FKYM01053337.1_30324:30354 | - | CCGGACATGGCCTGCACACGGTGCCCAGAAT | | 0 | 3 | 0 | 0 | 0 | 1 | 0 | 0 |
| Oe_mir_111 | FKYM01053185.1_34631:34658 | - | | CTTGGACCGTTGGATTGACCCGCTGATC | 0 | 19 | 0 | 0 | 0 | 3 | 0 | 0 |
| Oe_mir_112 | FKYM01053020.1_54191:54219 | + | | TTCAGGCCAAGCTCGTCGTCTTCACGGCG | 0 | 3 | 0 | 0 | 0 | 1 | 0 | 0 |
| Oe_mir_113 | FKYM01052486.1_51784:51811 | - | | AGACCGGAATTTGAAGCAGAGATGGAGA | 0 | 3 | 0 | 0 | 0 | 1 | 0 | 0 |
| Oe_mir_114 | FKYM01052372.1_12262:12292 | - | | CTTGAGGATGGTCGCACGTTGGCGGATTATA | 0 | 3 | 0 | 0 | 0 | 1 | 0 | 0 |
| Oe_mir_115 | FKYM01051749.1_34702:34732 | - | CTACTCGACCCCTTCTCTGAATTTTAACAGT | | 0 | 4 | 0 | 0 | 0 | 1 | 0 | 0 |
| Oe_mir_116 | FKYM01051150.1_96483:96510 | - | | TTATACCTATGAATTCCATTGGACCCAG | 0 | 4 | 0 | 0 | 0 | 1 | 0 | 0 |
| Oe_mir_117 | FKYM01050899.1_46311:46340 | + | | GCACACTGTGCGGCTCCATCGGCATGGCAA | 0 | 5 | 0 | 0 | 0 | 1 | 0 | 0 |
| Oe_mir_118 | FKYM01050454.1_146736:146763 | - | | CTTGGCGTGTAGCTGAAGCTCTTGAATA | 0 | 3 | 0 | 3 | 0 | 1 | 0 | 1 |
| Oe_mir_119 | FKYM01050453.1_4823:4854 | + | | TGGGCTGCAGAATGAAATGTTTGGAGATTCGA | 0 | 6 | 0 | 0 | 0 | 1 | 0 | 0 |
| Oe_mir_120 | FKYM01050353.1_28610:28638 | + | ACAGTGGATTGTTGTACTCACTGGCGAAT | | 0 | 3 | 0 | 0 | 0 | 1 | 0 | 0 |
| Oe_mir_121 | FKYM01049968.1_32946:32974 | + | | CAGGTGTGGTCATTGCAAAAGCCTTGCTC | 0 | 3 | 0 | 0 | 0 | 1 | 0 | 0 |
| Oe_mir_122 | FKYM01047776.1_5933:5961 | + | ATGTTATCTCACGTCTTGTTGGCAGCCGC | | 0 | 3 | 0 | 0 | 0 | 1 | 0 | 0 |
| Oe_mir_123 | FKYM01047533.1_1444:1472 | - | CGACAAGGACTTAATGGAAGGAAGTTTGG | | 0 | 5 | 0 | 0 | 0 | 1 | 0 | 0 |
| Oe_mir_124 | FKYM01047078.1_645:673 | + | | TTCCTCAATCGACAAAACTGTAAGAGTAT | 0 | 4 | 0 | 0 | 0 | 1 | 0 | 0 |
| Oe_mir_125 | FKYM01047065.1_42827:42857 | - | CTCAGATGGATGACAGCGGACATCGTGAAAA | | 0 | 3 | 0 | 0 | 0 | 1 | 0 | 0 |

| | | | | | | | | | | | | |
|---|---|---|---|---|---|---|---|---|---|---|---|---|
| Oe_mir_126 | FKYM01046860.1_475:502 | + | ATGGGCTAATTCATAGATCTTTGGGCTT | | 0 | 6 | 0 | 0 | 0 | 1 | 0 | 0 |
| Oe_mir_127 | FKYM01046529.1_40911:40940 | - | TTTAACCTTGGCTGGTGCTCTCGACCATGA | | 0 | 3 | 0 | 0 | 0 | 1 | 0 | 0 |
| Oe_mir_128 | FKYM01046256.1_65825:65854 | - | | CCTAAAGAGTAACGGAGGTGTGCGATGGCA | 0 | 5 | 0 | 0 | 0 | 1 | 0 | 0 |
| Oe_mir_129 | FKYM01046010.1_24247:24276 | - | | CTTGAAGAGTTATAGAATGTTCTGAGGCCG | 0 | 132 | 123 | 51 | 0 | 19 | 40 | 13 |
| Oe_mir_130 | FKYM01045508.1_19849:19877 | + | | GTTCACACACTCGACACTTGGCCGCCCCT | 0 | 4 | 0 | 0 | 0 | 1 | 0 | 0 |
| Oe_mir_131 | FKYM01045431.1_52605:52632 | + | | GCATCGTGTTTTGTAGTGTCTTCCCCTC | 0 | 3 | 0 | 0 | 0 | 1 | 0 | 0 |
| Oe_mir_132 | FKYM01045414.1_40636:40665 | - | | AACATCCTCGGGCCATTGACTAGCTTAAAA | 0 | 3 | 0 | 0 | 0 | 1 | 0 | 0 |
| Oe_mir_133 | FKYM01045316.1_9274:9303 | + | | ACCGACTGCTTGAAATTCCTCCAGTGGCCT | 0 | 3 | 0 | 0 | 0 | 1 | 0 | 0 |
| Oe_mir_134 | FKYM01045207.1_84621:84650 | + | GTTGACAATTAGACACATGAAGGGGGGCCC | | 0 | 3 | 0 | 0 | 0 | 1 | 0 | 0 |
| Oe_mir_135 | FKYM01045043.1_35421:35449 | - | | CAATTTGGCGTCTGGATATCTGAATTCTT | 0 | 3 | 0 | 0 | 0 | 1 | 0 | 0 |
| Oe_mir_136 | FKYM01044424.1_68296:68327 | + | | ACCAAAACTCTACCCTTCGGCCACCTTGGCTA | 0 | 4 | 0 | 0 | 0 | 1 | 0 | 0 |
| Oe_mir_137 | FKYM01043974.1_52593:52621 | - | | CATGTGCTCTCCACCCTGGCTCCTGGGAG | 0 | 3 | 0 | 0 | 0 | 1 | 0 | 0 |
| Oe_mir_138 | FKYM01043922.1_628:658 | + | | TTGTGAATGACATCTGGGGTTCCATCCCGCA | 0 | 13 | 0 | 0 | 0 | 2 | 0 | 0 |
| Oe_mir_139 | FKYM01043922.1_1250:1279 | + | | GGTGGAAATCGAGGTGGGGAGGTAATGGTG | 0 | 3 | 0 | 0 | 0 | 1 | 0 | 0 |
| Oe_mir_140 | FKYM01043625.1_36768:36798 | - | | CATCTGATGTTTCTGTACGCTGTTTAAGCCG | 0 | 3 | 0 | 0 | 0 | 1 | 0 | 0 |
| Oe_mir_141 | FKYM01043549.1_30443:30470 | + | | TGGAGAGTGCTGTTGCGGTAGCCGGACA | 0 | 4 | 0 | 0 | 0 | 1 | 0 | 0 |
| Oe_mir_142 | FKYM01043441.1_63848:63875 | + | GGTTATGAAGTAGTAACACTTGACGAAG | | 0 | 3 | 0 | 0 | 0 | 1 | 0 | 0 |
| Oe_mir_143 | FKYM01043315.1_12502:12530 | + | | AAAGTTGGTTTTGGTTTTCTTTGTTTAGT | 0 | 3 | 0 | 0 | 0 | 1 | 0 | 0 |
| Oe_mir_144 | FKYM01043295.1_24797:24828 | - | CATTCGACTAATTAGTGATCCCTGGGCCTACC | | 0 | 3 | 4 | 0 | 0 | 1 | 2 | 0 |
| Oe_mir_145 | FKYM01043227.1_12498:12526 | + | TCGTTTCATTAACTGTTGCTTTGAAAATT | | 0 | 4 | 0 | 0 | 0 | 1 | 0 | 0 |
| Oe_mir_146 | FKYM01042830.1_16146:16175 | + | GCTTTATAGATCTCCATGATATAGTCGTTC | | 0 | 3 | 0 | 0 | 0 | 1 | 0 | 0 |
| Oe_mir_147 | FKYM01042502.1_67650:67677 | - | TTTGTGGTAGGGCATTTCTGCAAGCGTT | | 0 | 4 | 0 | 0 | 0 | 1 | 0 | 0 |
| Oe_mir_148 | FKYM01042085.1_1948:1978 | + | AAGGAGAGAAGTCGATGGGTACCATGCTTTT | | 0 | 3 | 0 | 0 | 0 | 1 | 0 | 0 |
| Oe_mir_149 | FKYM01042061.1_7863:7891 | + | TGTCATCACCGAGCTGGGCCGTCGAGCGA | | 0 | 3 | 0 | 0 | 0 | 1 | 0 | 0 |
| Oe_mir_150 | FKYM01042027.1_9590:9619 | - | | TCGAACCGAACCGACCGAACCGAACCCGAC | 0 | 3 | 0 | 0 | 0 | 1 | 0 | 0 |
| Oe_mir_151 | FKYM01041892.1_581:608 | - | | CGCCGATAACTTGATATATTTAAGGTAC | 0 | 5 | 0 | 0 | 0 | 1 | 0 | 0 |
| Oe_mir_152 | FKYM01041563.1_61699:61729 | - | CCCTTGAAAATCCGAAGGATCGAGTACCGTC | | 0 | 5 | 0 | 0 | 0 | 1 | 0 | 0 |
| Oe_mir_153 | FKYM01041310.1_64778:64809 | + | AGAGTTTCTTAGAGCCACTGCCCTTGCGGCTG | | 0 | 3 | 0 | 0 | 0 | 1 | 0 | 0 |
| Oe_mir_154 | FKYM01041219.1_14158:14186 | - | | CCAGGGCGGCCACCTCGAAATCTTCCGGT | 0 | 3 | 0 | 0 | 0 | 1 | 0 | 0 |
| Oe_mir_155 | FKYM01041150.1_361:392 | - | | CTTTGGTTTATTCGACTCCACCACTCCTCTCA | 0 | 13 | 0 | 0 | 0 | 2 | 0 | 0 |
| Oe_mir_156 | FKYM01041092.1_51841:51868 | + | ACGCCTACTCTTTCGTTCGGGATTATGA | | 0 | 3 | 0 | 0 | 0 | 1 | 0 | 0 |
| Oe_mir_157 | FKYM01040974.1_60508:60538 | + | TTGGCTCGACTCGGAGATGAACTACACTTTT | | 0 | 3 | 0 | 0 | 0 | 1 | 0 | 0 |

| | | | | | | | | | | | | |
|---|---|---|---|---|---|---|---|---|---|---|---|---|
| Oe_mir_158 | FKYM01040944.1_21036:21067 | + | | GGTCGTAGGTTCGAATCCTACTTGGGGAGATT | 0 | 10 | 0 | 0 | 0 | 2 | 0 | 0 |
| Oe_mir_159 | FKYM01040758.1_3058:3085 | - | CCCGTGGTGGAAGCAGATTTCTGGGCCC | | 0 | 3 | 0 | 0 | 0 | 1 | 0 | 0 |
| Oe_mir_160 | FKYM01040472.1_43462:43492 | - | | CTGAACTTTGCCGAAATTCCCTAGAGAGGTC | 0 | 12 | 0 | 0 | 0 | 2 | 0 | 0 |
| Oe_mir_161 | FKYM01039660.1_42762:42789 | - | | CTCGTGACTCAATTGGAGAACTGATGCA | 0 | 3 | 0 | 0 | 0 | 1 | 0 | 0 |
| Oe_mir_162 | FKYM01039644.1_34763:34794 | + | | ATGTGCTCTCCACCCTGGCTCCTGGGAGGCAC | 0 | 7 | 0 | 0 | 0 | 1 | 0 | 0 |
| Oe_mir_163 | FKYM01039644.1_33671:33700 | - | | GAGTTGACACCTCGGATGACACTCTACACA | 0 | 3 | 0 | 0 | 0 | 1 | 0 | 0 |
| Oe_mir_164 | FKYM01038462.1_1224:1254 | + | TGGTTGATCCTGCCAGTAGTCATATGCTTGT | | 0 | #### | 0 | 0 | 0 | 249 | 0 | 0 |
| Oe_mir_165 | FKYM01037829.1_36200:36228 | + | | AGAAGAAAGATGCAGGATTTGATGGGCAA | 0 | 3 | 0 | 0 | 0 | 1 | 0 | 0 |
| Oe_mir_166 | FKYM01037652.1_32745:32772 | + | GCCCTGCTGCTCGAGACGAAGAGCTCAA | | 0 | 3 | 0 | 0 | 0 | 1 | 0 | 0 |
| Oe_mir_167 | FKYM01037411.1_2451:2481 | + | AGACTATCTAGTTGTTGAACGCGCTCCTTAT | | 0 | 4 | 0 | 0 | 0 | 1 | 0 | 0 |
| Oe_mir_168 | FKYM01037342.1_7411:7442 | + | AAAGGAGAGGAGGATGAGGACTTTCAAATTTT | | 0 | 3 | 3 | 0 | 0 | 1 | 1 | 0 |
| Oe_mir_169 | FKYM01036609.1_19878:19908 | + | | TTTGCATAAACTCTCACATCTCCAAGGACTT | 0 | 3 | 0 | 0 | 0 | 1 | 0 | 0 |
| Oe_mir_170 | FKYM01036575.1_61827:61855 | - | CCTCTTTGAAATGGGCTAGGGCTAAAACA | | 0 | 3 | 0 | 0 | 0 | 1 | 0 | 0 |
| Oe_mir_171 | FKYM01036452.1_35378:35406 | - | CTAATGAGAGCCACTGATGTGATGATTGC | | 0 | 4 | 0 | 0 | 0 | 1 | 0 | 0 |
| Oe_mir_172 | FKYM01036277.1_2975:3005 | - | CACCCCCATGTGAGAGGCCCTGTACAGACCC | | 0 | 3 | 0 | 0 | 0 | 1 | 0 | 0 |
| Oe_mir_173 | FKYM01036130.1_1873:1901 | + | GACTTGGGATCATGTCTTCTTAGGACTAT | | 0 | 5 | 0 | 0 | 0 | 1 | 0 | 0 |
| Oe_mir_174 | FKYM01035677.1_10375:10403 | + | | CTGTGATGAATTTTTGAATCAGAAGATCA | 0 | 7 | 0 | 0 | 0 | 1 | 0 | 0 |
| Oe_mir_175 | FKYM01035436.1_188744:188773 | + | | GTGGCCAAGTGGTAAGGCAACGGGTTTTGG | 0 | 11 | 0 | 0 | 0 | 2 | 0 | 0 |
| Oe_mir_176 | FKYM01035104.1_61715:61743 | - | CACTGTACAATCGGACATTGAAATTTGCG | | 0 | 3 | 0 | 0 | 0 | 1 | 0 | 0 |
| Oe_mir_177 | FKYM01035061.1_32346:32376 | - | | TTGTTGGTCTTCAACGAGGAATTCCTAGTAA | 0 | 14 | 0 | 0 | 0 | 2 | 0 | 0 |
| Oe_mir_178 | FKYM01035061.1_32122:32150 | - | TGGAAGGATCATTGTCGAAACCTACAAAA | | 0 | 4 | 0 | 0 | 0 | 1 | 0 | 0 |
| Oe_mir_179 | FKYM01035043.1_111590:111617 | + | AATGGGGTGCAGGAGGAATTGGTGCAAT | | 0 | 3 | 0 | 0 | 0 | 1 | 0 | 0 |
| Oe_mir_180 | FKYM01034952.1_10057:10085 | + | | GTTGTTTGTTGGATCTGAAATTTTGAAAT | 0 | 4 | 0 | 0 | 0 | 1 | 0 | 0 |
| Oe_mir_181 | FKYM01034511.1_156768:156798 | - | | CCCTTCGAGCAGGATATGCTATCTTTGCGTC | 0 | 8 | 0 | 5 | 0 | 1 | 0 | 2 |
| Oe_mir_182 | FKYM01034152.1_1289:1320 | + | | CACCTTGGCTGCCGGACCGACATACCACTTTG | 0 | 3 | 0 | 0 | 0 | 1 | 0 | 0 |
| Oe_mir_183 | FKYM01034081.1_29017:29045 | + | GAAGATTCTGTGACAACATCGTAGGATAC | | 0 | 3 | 0 | 0 | 0 | 1 | 0 | 0 |
| Oe_mir_184 | FKYM01033814.1_6356:6387 | + | TTTAGATCTGAACGGACTGGTAAATTTGGGCC | | 0 | 3 | 0 | 0 | 0 | 1 | 0 | 0 |
| Oe_mir_185 | FKYM01033808.1_27257:27284 | - | TATGTTGTGGTGGCGTTAAGAATTCATT | | 0 | 4 | 0 | 0 | 0 | 1 | 0 | 0 |
| Oe_mir_186 | FKYM01033761.1_269336:269364 | + | ACCCAAACTACTCCAATCAACCCGACTCG | | 0 | 3 | 0 | 0 | 0 | 1 | 0 | 0 |
| Oe_mir_187 | FKYM01033663.1_137754:137784 | - | ACCTTCGCCTTTTACCACTTGGAGATGTGCC | | 0 | 3 | 0 | 0 | 0 | 1 | 0 | 0 |
| Oe_mir_188 | FKYM01033662.1_5592:5620 | + | | TCGTGAGGACCGTATTGGTGTTTGCAAGG | 0 | 3 | 0 | 0 | 0 | 1 | 0 | 0 |
| Oe_mir_189 | FKYM01032959.1_25750:25777 | - | TATGATGATTTGTCGATGGGAATCTCTC | | 0 | 9 | 0 | 0 | 0 | 1 | 0 | 0 |

| | | | | | | | | | | | | |
|---|---|---|---|---|---|---|---|---|---|---|---|---|
| Oe_mir_190 | FKYM01032593.1_39528:39558 | + | | AGAATGTTTTGGCTGCTTGTAACTTTGATTT | 0 | 3 | 0 | 0 | 0 | 1 | 0 | 0 |
| Oe_mir_191 | FKYM01032455.1_173952:173983 | - | | CTGACATTGATTCCACTGATTGTTGCTTCCAG | 0 | 9 | 0 | 0 | 0 | 1 | 0 | 0 |
| Oe_mir_192 | FKYM01032398.1_38958:38986 | - | CTTTGAGTTCTTGGGAAACCTTAGTGGGG | | 0 | 3 | 0 | 0 | 0 | 1 | 0 | 0 |
| Oe_mir_193 | FKYM01032261.1_34412:34439 | + | GCTTGGGCTAAGATATATGGAACCTCGA | | 0 | 3 | 0 | 0 | 0 | 1 | 0 | 0 |
| Oe_mir_194 | FKYM01032181.1_16889:16920 | - | CTGAACTGCTGGCTGAGAAGAGATGTGTAGCT | | 0 | 3 | 0 | 0 | 0 | 1 | 0 | 0 |
| Oe_mir_195 | FKYM01032104.1_25851:25882 | + | ACTGGATCAAGGTCTGGATTATGTCGCCACAT | | 0 | 6 | 0 | 3 | 0 | 1 | 0 | 1 |
| Oe_mir_196 | FKYM01031956.1_33577:33607 | + | | AAGATGGGTACACTCAAGATGGGACTGTAGA | 0 | 3 | 0 | 0 | 0 | 1 | 0 | 0 |
| Oe_mir_197 | FKYM01031715.1_553:583 | - | CCCGCCTGGGGAGTACGTTTGCAAGAATGAA | | 0 | 3 | 0 | 0 | 0 | 1 | 0 | 0 |
| Oe_mir_198 | FKYM01031697.1_32005:32032 | - | | CTGGCATACTTTGATCATGGAGGCATCC | 0 | 4 | 0 | 0 | 0 | 1 | 0 | 0 |
| Oe_mir_199 | FKYM01031615.1_24175:24202 | + | | CTTAACTTTTGAAATTAGAGTTTGACTC | 0 | 3 | 0 | 0 | 0 | 1 | 0 | 0 |
| Oe_mir_200 | FKYM01031548.1_41101:41128 | - | CTCTTCTTCCCAAATCTCTCTGCGTGAA | | 0 | 3 | 0 | 0 | 0 | 1 | 0 | 0 |
| Oe_mir_201 | FKYM01031303.1_105321:105351 | + | | AATCAGTACATGAACCCCCGTCCTTTAGAAA | 0 | 3 | 0 | 0 | 0 | 1 | 0 | 0 |
| Oe_mir_202 | FKYM01030905.1_721:748 | + | | GGCTCATACAGAACCGTTGAAGATATAT | 0 | 4 | 0 | 0 | 0 | 1 | 0 | 0 |
| Oe_mir_203 | FKYM01030870.1_51900:51928 | + | | CGTTGTTGGAGTTGGATCTCAGTGGCAAT | 0 | 7 | 4 | 0 | 0 | 1 | 2 | 0 |
| Oe_mir_204 | FKYM01030824.1_348:375 | - | CGCCTCCGCTTATTCTGTATCGGTACAC | | 0 | 4 | 0 | 0 | 0 | 1 | 0 | 0 |
| Oe_mir_205 | FKYM01030424.1_7122:7152 | + | AGAAAAAGAAGAAGAAGAGAAAGAAAGAAAT | | 0 | 3 | 0 | 0 | 0 | 1 | 0 | 0 |
| Oe_mir_206 | FKYM01030327.1_64018:64047 | + | AGGGCAGAAGAGAATGATTGAACTTGGTAT | | 0 | 3 | 0 | 0 | 0 | 1 | 0 | 0 |
| Oe_mir_207 | FKYM01030115.1_2788:2817 | - | | CTGCCCATTGAGATAAGAGATCAAGATTGT | 0 | 3 | 0 | 0 | 0 | 1 | 0 | 0 |
| Oe_mir_208 | FKYM01029967.1_18286:18315 | + | | ATACCTTTTGTTCCACTTCCAGTTCCTATG | 0 | 5 | 0 | 0 | 0 | 1 | 0 | 0 |
| Oe_mir_209 | FKYM01028810.1_612:642 | - | | CTAGGAAGAGCAGCATACTTGGAGGATTGGC | 0 | 3 | 0 | 0 | 0 | 1 | 0 | 0 |
| Oe_mir_210 | FKYM01028100.1_2654:2684 | + | AAATCAAACAGGGGCTTCTGTAGATTGGGCT | | 0 | 3 | 0 | 0 | 0 | 1 | 0 | 0 |
| Oe_mir_211 | FKYM01027885.1_29180:29209 | - | ACAATGGAATCAGTTTCTTAGGAAGGCCAC | | 0 | 7 | 0 | 0 | 0 | 1 | 0 | 0 |
| Oe_mir_212 | FKYM01027527.1_7775:7802 | + | AACGTTACTGTTATTATCATGAACAAAT | | 0 | 4 | 0 | 0 | 0 | 1 | 0 | 0 |
| Oe_mir_213 | FKYM01027278.1_291:318 | - | TGTTAAATCTATTTCCTGCACATTGGTT | | 0 | 3 | 0 | 0 | 0 | 1 | 0 | 0 |
| Oe_mir_214 | FKYM01026684.1_21524:21551 | - | GTAAGCCATGTCTCTTCATTGAGGTCGC | | 0 | 4 | 0 | 0 | 0 | 1 | 0 | 0 |
| Oe_mir_215 | FKYM01026603.1_7850:7877 | - | | TGGATGTGAGAGATAAAAATAAAGGGTT | 0 | 3 | 0 | 0 | 0 | 1 | 0 | 0 |
| Oe_mir_216 | FKYM01026590.1_3110:3137 | + | CATTTACTCGTCTCATTGTACACTAATG | | 0 | 4 | 0 | 0 | 0 | 1 | 0 | 0 |
| Oe_mir_217 | FKYM01026399.1_2058:2089 | + | TAAGATTAGAGCGCATGCTCAAGTTGTTCGGG | | 0 | 5 | 0 | 0 | 0 | 1 | 0 | 0 |
| Oe_mir_218 | FKYM01026253.1_22880:22909 | + | GGTGTCCCGTTCAGTCAAAGCCGGTCTGCA | | 0 | 3 | 0 | 0 | 0 | 1 | 0 | 0 |
| Oe_mir_219 | FKYM01026244.1_11719:11748 | + | AGTGGCAAGAAAAGGACAGTCGAGAAGAGA | | 0 | 3 | 0 | 0 | 0 | 1 | 0 | 0 |
| Oe_mir_220 | FKYM01026217.1_6151:6179 | + | | TTGTCAGTTCGTGGTGCTGTTACTCATTT | 0 | 4 | 0 | 0 | 0 | 1 | 0 | 0 |
| Oe_mir_221 | FKYM01026167.1_1447:1475 | - | TATCGACACCTAAGATTGATGACCAGACC | | 0 | 3 | 0 | 0 | 0 | 1 | 0 | 0 |

| | | | | | | | | | | | | |
|---|---|---|---|---|---|---|---|---|---|---|---|---|
| Oe_mir_222 | FKYM01025872.1_11227:11257 | + | | TTGGTCATCTGGGCGTTTGTAGATCAAGAAT | 0 | 4 | 0 | 0 | 0 | 1 | 0 | 0 |
| Oe_mir_223 | FKYM01025820.1_24211:24239 | - | CAGTATTAGACGCCCCAGAAACTGAAGGA | | 0 | 4 | 0 | 3 | 0 | 1 | 0 | 1 |
| Oe_mir_224 | FKYM01025779.1_287:315 | - | | CGTTGGAATGCAAATGGTCGTCGAACCTT | 0 | 3 | 0 | 0 | 0 | 1 | 0 | 0 |
| Oe_mir_225 | FKYM01025563.1_59213:59240 | + | CTTTGTCTTTGGGGCTTACAATTCACCT | | 0 | 14 | 0 | 0 | 0 | 2 | 0 | 0 |
| Oe_mir_226 | FKYM01025129.1_10921:10952 | - | | GGGTATCTTGAAAGTCTCGGGTTCGAACCTTT | 0 | 3 | 0 | 0 | 0 | 1 | 0 | 0 |
| Oe_mir_227 | FKYM01025050.1_77572:77602 | + | CCTCTGGCTGTCGCGTGCTTGTGAACCCTTG | | 0 | 4 | 0 | 0 | 0 | 1 | 0 | 0 |
| Oe_mir_228 | FKYM01024992.1_111314:111342 | + | | GGTAGGCTTTAGTCATTGGCGCTCGAGTG | 0 | 4 | 0 | 0 | 0 | 1 | 0 | 0 |
| Oe_mir_229 | FKYM01024767.1_64355:64384 | - | | CACTCACCACCACCTTTCCAATTCATTGCA | 0 | 3 | 0 | 0 | 0 | 1 | 0 | 0 |
| Oe_mir_230 | FKYM01023731.1_32271:32298 | + | CATGGGCCAGGCTTGATGCGAACCGGCC | | 0 | 4 | 0 | 0 | 0 | 1 | 0 | 0 |
| Oe_mir_231 | FKYM01023446.1_7389:7418 | + | | TTTGCAGGATGGGGAACAAATGAGAAATTG | 0 | 3 | 0 | 0 | 0 | 1 | 0 | 0 |
| Oe_mir_232 | FKYM01023343.1_42295:42322 | - | | GGGGCTGAAGGATCATGAAGACTTGATT | 0 | 3 | 0 | 0 | 0 | 1 | 0 | 0 |
| Oe_mir_233 | FKYM01023310.1_82754:82781 | + | | CTTTCATGGTGTAGTTCACATTGCTCCA | 0 | 4 | 0 | 0 | 0 | 1 | 0 | 0 |
| Oe_mir_234 | FKYM01022965.1_14715:14742 | - | ATTATTACTGCTGCTACTACTACTTGAC | | 0 | 3 | 0 | 0 | 0 | 1 | 0 | 0 |
| Oe_mir_235 | FKYM01022919.1_11172:11200 | + | | ATTGGCGGAGGCTGATGATAATGGGAGCA | 0 | 3 | 0 | 0 | 0 | 1 | 0 | 0 |
| Oe_mir_236 | FKYM01022889.1_105454:105483 | - | GGAGAGACTGAGTGCTCAGTTATCATGTTC | | 0 | 4 | 5 | 0 | 0 | 1 | 2 | 0 |
| Oe_mir_237 | FKYM01022630.1_148806:148834 | - | | CGAGTGGGGATTGGTACTGATGGTTTCAT | 0 | 3 | 0 | 0 | 0 | 1 | 0 | 0 |
| Oe_mir_238 | FKYM01022360.1_18270:18298 | - | GCCCTGGGAAGTCCTCGTGTTGCACCCCT | | 0 | 149 | 0 | 0 | 0 | 21 | 0 | 0 |
| Oe_mir_239 | FKYM01022360.1_21839:21868 | - | GTTAAGCGTGCTTGGGCGAGAGTAGTACTA | | 0 | 6 | 0 | 0 | 0 | 1 | 0 | 0 |
| Oe_mir_240 | FKYM01022359.1_6030:6057 | - | | CTTGGGCGAGAGTAGTACTAGGATGGAT | 0 | 6 | 0 | 0 | 0 | 1 | 0 | 0 |
| Oe_mir_241 | FKYM01022358.1_21056:21086 | + | | TTAACTTCGGAGTTCTGATGGGATCCGAGGC | 0 | 3 | 0 | 0 | 0 | 1 | 0 | 0 |
| Oe_mir_242 | FKYM01022358.1_10020:10047 | - | CCCCTGGGAAGTCCTCGTGTTGCACCCC | | 0 | #### | 0 | 0 | 0 | 406 | 0 | 0 |
| Oe_mir_243 | FKYM01022358.1_10678:10706 | - | | CCCCTGGGAAGTCCTCGTGTTGCCCCCCT | 0 | 33 | 0 | 0 | 0 | 5 | 0 | 0 |
| Oe_mir_244 | FKYM01022358.1_22439:22467 | - | | CCCGTGGGAAGTCCTCGTGTTGCACCCCT | 0 | 17 | 0 | 0 | 0 | 3 | 0 | 0 |
| Oe_mir_245 | FKYM01022012.1_27180:27211 | + | GACTAGTCTTGTTTGGTTGTATTTGCTCTTTG | | 0 | 3 | 0 | 0 | 0 | 1 | 0 | 0 |
| Oe_mir_246 | FKYM01021721.1_37399:37428 | + | | TACTGAGACGCAGCACTGGAGATTTATGAT | 0 | 4 | 0 | 0 | 0 | 1 | 0 | 0 |
| Oe_mir_247 | FKYM01021364.1_134468:134498 | + | | CTCCATTCACAGACTTCCTGTTTTGTACCAA | 0 | 3 | 0 | 0 | 0 | 1 | 0 | 0 |
| Oe_mir_248 | FKYM01021342.1_14557:14585 | - | | CTTTGTTCGTTGATTTTGTCTGTTTGCAG | 0 | 3 | 0 | 0 | 0 | 1 | 0 | 0 |
| Oe_mir_249 | FKYM01020992.1_55162:55190 | - | | TAGGCTGGTTCGGATTGGTTATACATAGT | 0 | 4 | 0 | 0 | 0 | 1 | 0 | 0 |
| Oe_mir_250 | FKYM01020931.1_11967:11998 | - | | ATTGTACACACCGCCCGTCACACTATGGGAGC | 0 | 15 | 0 | 0 | 0 | 2 | 0 | 0 |
| Oe_mir_251 | FKYM01020887.1_50338:50365 | - | | TTCTCCTTGCTTTGATTGCAGTATCTGT | 0 | 3 | 0 | 0 | 0 | 1 | 0 | 0 |
| Oe_mir_252 | FKYM01020836.1_54001:54029 | - | | CTGGATTTAGAACAAAACAAGAGACAGCC | 0 | 4 | 0 | 0 | 0 | 1 | 0 | 0 |
| Oe_mir_253 | FKYM01020806.1_47918:47946 | + | | GCTACATTTTTACATTTTCTTGTTGGATA | 0 | 5 | 0 | 0 | 0 | 1 | 0 | 0 |

| | | | | | | | | | | | |
|---|---|---|---|---|---|---|---|---|---|---|---|
| Oe_mir_254 | FKYM01020739.1_18088:18118 | - | CTGGGTCATGAAATTGAGAATATTGTAGAGC | | 0 | 3 | 0 | 0 | 0 | 1 | 0 | 0 |
| Oe_mir_255 | FKYM01020737.1_19863:19892 | + | AACATCAGCTGCTGCTTCTCCCATGGGGGG | | 0 | 3 | 0 | 0 | 0 | 1 | 0 | 0 |
| Oe_mir_256 | FKYM01020655.1_53523:53550 | - | | CAAGTGGAATTGTGTTGACAGTTGCTGT | 0 | 7 | 3 | 0 | 0 | 1 | 1 | 0 |
| Oe_mir_257 | FKYM01020576.1_26883:26914 | - | | CTGCCTGCCAATGTTGCTGATCTCCTGTAAGG | 0 | 4 | 0 | 0 | 0 | 1 | 0 | 0 |
| Oe_mir_258 | FKYM01020304.1_32011:32038 | - | | CCTGGACCTGTTTGATAACTTGATGTAG | 0 | 3 | 0 | 0 | 0 | 1 | 0 | 0 |
| Oe_mir_259 | FKYM01020216.1_29780:29808 | - | CCAGTGGTCTAGTGGTAGAATAGTACCCT | | 0 | 74 | 0 | 0 | 0 | 10 | 0 | 0 |
| Oe_mir_260 | FKYM01020215.1_15652:15679 | - | | CGTGGCCAAGTGGTAAGGCAACGGGTTT | 0 | 72 | 0 | 15 | 0 | 10 | 0 | 4 |
| Oe_mir_261 | FKYM01019908.1_177537:177565 | - | | CTGTCTGGATGGATATATGAATATAGCGA | 0 | 3 | 0 | 0 | 0 | 1 | 0 | 0 |
| Oe_mir_262 | FKYM01019888.1_34696:34724 | + | | TAGGCATCGATGGATGGGACTGATTTGAA | 0 | 5 | 0 | 0 | 0 | 1 | 0 | 0 |
| Oe_mir_263 | FKYM01018286.1_1424:1453 | - | | CATTTGTGGAGGAACAAGAACTTGCTTATT | 0 | 3 | 0 | 0 | 0 | 1 | 0 | 0 |
| Oe_mir_264 | FKYM01018039.1_6767:6795 | - | | CTGTGAGTCGGGACTTGCTAAGGTCAATC | 0 | 3 | 0 | 0 | 0 | 1 | 0 | 0 |
| Oe_mir_265 | FKYM01017651.1_96711:96738 | + | | GAGAAGGAATTAGCTGCTGTTAAGATTA | 0 | 3 | 0 | 0 | 0 | 1 | 0 | 0 |
| Oe_mir_266 | FKYM01017270.1_1650:1677 | + | CAACAGATCGTCGCATTTTAGTCCTTCT | | 0 | 3 | 0 | 0 | 0 | 1 | 0 | 0 |
| Oe_mir_267 | FKYM01016520.1_1151:1179 | - | CAATTACCAGACTCTGAGAGCCCGGTATT | | 0 | 4 | 0 | 0 | 0 | 1 | 0 | 0 |
| Oe_mir_268 | FKYM01016251.1_16190:16220 | + | | AGGGAATAAGAGCTGTCTGTACACTGCGCCT | 0 | 3 | 0 | 0 | 0 | 1 | 0 | 0 |
| Oe_mir_269 | FKYM01016140.1_893:921 | - | ATGAGAGAGAAAAATGAAATGTAATAAAA | | 0 | 3 | 0 | 0 | 0 | 1 | 0 | 0 |
| Oe_mir_270 | FKYM01016107.1_50797:50825 | - | | AGGGTATGCAAAATGAACACTCGGCGCCC | 0 | 3 | 0 | 0 | 0 | 1 | 0 | 0 |
| Oe_mir_271 | FKYM01015889.1_3257:3286 | - | CAGGTTGTTGGAGGCAGCAGATGAGAGGAT | | 0 | 3 | 0 | 0 | 0 | 1 | 0 | 0 |
| Oe_mir_272 | FKYM01015691.1_7881:7909 | + | | TAGTGTGGACGTTGGAGATTTAGTAAGTC | 0 | 6 | 0 | 0 | 0 | 1 | 0 | 0 |
| Oe_mir_273 | FKYM01015362.1_13420:13447 | - | TTTTGTCTTCTGATGTCTTTTGATGCAG | | 0 | 3 | 0 | 0 | 0 | 1 | 0 | 0 |
| Oe_mir_274 | FKYM01015154.1_43247:43275 | - | TCTGTACCGATTGAACTTTGCTACTTGTA | | 0 | 4 | 0 | 0 | 0 | 1 | 0 | 0 |
| Oe_mir_275 | FKYM01014890.1_29876:29905 | - | | GGTGAAGCGGAGGAAGTTTGGACTAAGGAG | 0 | 4 | 0 | 0 | 0 | 1 | 0 | 0 |
| Oe_mir_276 | FKYM01014814.1_23059:23086 | + | | TCTGAACTGAGAACACAGGCAGATTTAT | 0 | 3 | 0 | 0 | 0 | 1 | 0 | 0 |
| Oe_mir_277 | FKYM01014745.1_573:601 | + | TCTCTAGACTGTGTCGGAATTGGCTGAAA | | 0 | 3 | 0 | 0 | 0 | 1 | 0 | 0 |
| Oe_mir_278 | FKYM01014521.1_2636:2664 | + | | GTATTGATGTGTAGCTTGGAGGAGTTGTA | 0 | 4 | 0 | 0 | 0 | 1 | 0 | 0 |
| Oe_mir_279 | FKYM01014450.1_36446:36473 | + | CTCCATACTGACGAACCATCTGTTGATG | | 0 | 3 | 0 | 0 | 0 | 1 | 0 | 0 |
| Oe_mir_280 | FKYM01014275.1_38979:39006 | + | | ATTTGGTCTAGTGGTATGATTCTCGCTT | 0 | 61 | 0 | 0 | 0 | 9 | 0 | 0 |
| Oe_mir_281 | FKYM01014275.1_39321:39349 | - | | CATTTGGTCTAGTGGTATGATTCTCGCTT | 0 | 13 | 0 | 0 | 0 | 2 | 0 | 0 |
| Oe_mir_282 | FKYM01014226.1_36524:36552 | + | TGATGGAACTGGCGAAGGACGGTGACGGT | | 0 | 3 | 0 | 0 | 0 | 1 | 0 | 0 |
| Oe_mir_283 | FKYM01014067.1_11352:11380 | + | | ACCAGCGTTCCGATTGCTTCACATGTCTC | 0 | 6 | 0 | 0 | 0 | 1 | 0 | 0 |
| Oe_mir_284 | FKYM01014066.1_42739:42770 | + | | ATTTTTTCTATCCAACCCACAACTCATAAGTA | 0 | 3 | 0 | 0 | 0 | 1 | 0 | 0 |
| Oe_mir_285 | FKYM01013831.1_112003:112031 | + | | GGATTGAGGCGAGGGGAGAGGACGATAAT | 0 | 3 | 0 | 0 | 0 | 1 | 0 | 0 |

| | | | | | | | | | | | | |
|---|---|---|---|---|---|---|---|---|---|---|---|---|
| Oe_mir_286 | FKYM01013681.1_43255:43283 | + | AGAAGCTCTGACCCTAATAATCCGCAAAG | | 0 | 3 | 0 | 0 | 0 | 1 | 0 | 0 |
| Oe_mir_287 | FKYM01013460.1_48785:48813 | + | GTGGTTGAAAACACTGAGAGGGGATGGCG | | 0 | 3 | 0 | 0 | 0 | 1 | 0 | 0 |
| Oe_mir_288 | FKYM01012939.1_24989:25019 | + | TGGACCAAAGAGGTGGACTATAACGCGCAGA | | 0 | 3 | 0 | 0 | 0 | 1 | 0 | 0 |
| Oe_mir_289 | FKYM01012927.1_2686:2717 | - | CTGAGATTGTCGTTGGCCTTATTGATCACCTA | | 0 | 3 | 0 | 0 | 0 | 1 | 0 | 0 |
| Oe_mir_290 | FKYM01012386.1_11060:11089 | - | | CAGAGCAGATTCACGTCACAGGTTCAAATC | 0 | 3 | 0 | 0 | 0 | 1 | 0 | 0 |
| Oe_mir_291 | FKYM01012275.1_39013:39041 | + | TCTAGTTCGCAAAGGCCTGGAAAGAACTG | | 0 | 3 | 0 | 0 | 0 | 1 | 0 | 0 |
| Oe_mir_292 | FKYM01012177.1_74001:74029 | + | TGGAACGCCGTCGCGCTCTGGGCTTGGGG | | 0 | 5 | 0 | 0 | 0 | 1 | 0 | 0 |
| Oe_mir_293 | FKYM01011956.1_31804:31832 | + | GTGGAGGATTAGATCCAGTAACTGGGGGT | | 0 | 3 | 0 | 0 | 0 | 1 | 0 | 0 |
| Oe_mir_294 | FKYM01011839.1_22220:22247 | + | GAGACCGAGAAGTTGCTTTAGCTGACTC | | 0 | 3 | 0 | 0 | 0 | 1 | 0 | 0 |
| Oe_mir_295 | FKYM01011479.1_96921:96952 | - | CATGTGATGATTTGCTGGATATCTAGATGGCT | | 0 | 10 | 4 | 0 | 0 | 2 | 2 | 0 |
| Oe_mir_296 | FKYM01011318.1_22461:22489 | - | CTACATCGTATTGGACGTAGTGGGCGATT | | 0 | 20 | 0 | 0 | 0 | 3 | 0 | 0 |
| Oe_mir_297 | FKYM01011217.1_3441:3472 | + | | TTTTATGTCTTGATGAGATTGTTGATGGAGGG | 0 | 6 | 0 | 0 | 0 | 1 | 0 | 0 |
| Oe_mir_298 | FKYM01010646.1_44324:44355 | - | | GGCTGGCTCGAAGCAATCACAGTTTGCTGTTA | 0 | 9 | 0 | 2 | 0 | 1 | 0 | 1 |
| Oe_mir_299 | FKYM01010646.1_44283:44313 | - | TGTGAAGGCATCGGACCAGGCTTCATTCCTC | | 0 | 6 | 0 | 3 | 0 | 1 | 0 | 1 |
| Oe_mir_300 | FKYM01010420.1_5154:5184 | - | CAGGAGATGATGACACTGGCGGCGGAAGAGA | | 0 | 4 | 0 | 0 | 0 | 1 | 0 | 0 |
| Oe_mir_301 | FKYM01010102.1_550:579 | - | CTTGTTTCTGATGATGACGAAACACATATG | | 0 | 11 | 0 | 0 | 0 | 2 | 0 | 0 |
| Oe_mir_302 | FKYM01010006.1_13689:13718 | - | | CATTGGGCTAGAATTACTTGTGAGGGATTG | 0 | 4 | 0 | 0 | 0 | 1 | 0 | 0 |
| Oe_mir_303 | FKYM01009893.1_35512:35542 | - | | CAACGCGGGCAAATCGGAACTCTACTTGCCA | 0 | 3 | 0 | 0 | 0 | 1 | 0 | 0 |
| Oe_mir_304 | FKYM01009841.1_22720:22750 | - | | CTACTGCTTCCTAATCCATTGAAGTCAGATG | 0 | 3 | 0 | 0 | 0 | 1 | 0 | 0 |
| Oe_mir_305 | FKYM01009823.1_3172:3201 | + | | ATGCCTATGGAAATGTGGACCGATAAACCA | 0 | 5 | 0 | 0 | 0 | 1 | 0 | 0 |
| Oe_mir_306 | FKYM01008166.1_3621:3651 | - | | TTTTGGGTTACCCGACCCCACCACTCCTCTC | 0 | 5 | 0 | 0 | 0 | 1 | 0 | 0 |
| Oe_mir_307 | FKYM01007534.1_28559:28588 | - | TTTTCATTTTCACTTTTCTTATCGTTCACA | | 0 | 3 | 0 | 0 | 0 | 1 | 0 | 0 |
| Oe_mir_308 | FKYM01007076.1_17287:17316 | + | GGTGAGGCTGGGCGACCTGATGAGGTGGCA | | 0 | 35 | 15 | 0 | 0 | 5 | 5 | 0 |
| Oe_mir_309 | FKYM01006718.1_20647:20676 | - | CTCTTGGCGAATGTTTTGTAGATTCTGGAT | | 0 | 3 | 0 | 0 | 0 | 1 | 0 | 0 |
| Oe_mir_310 | FKYM01006580.1_94466:94497 | - | | CTTAAGAAAGCAATTCCGGCTCCGGCGATAAC | 0 | 3 | 0 | 0 | 0 | 1 | 0 | 0 |
| Oe_mir_311 | FKYM01006545.1_48432:48460 | + | CTTCAGGACTGTGAGAAGTGTGTATGAGT | | 0 | 6 | 0 | 0 | 0 | 1 | 0 | 0 |
| Oe_mir_312 | FKYM01006022.1_8662:8693 | + | | GTCGCGAAGCCCGATTTGCAGATTCCTGGCCG | 0 | 3 | 0 | 0 | 0 | 1 | 0 | 0 |
| Oe_mir_313 | FKYM01005955.1_4763:4791 | + | | GGGGTGAACGGCCGTAAGTTCCTGGTCCC | 0 | 3 | 0 | 0 | 0 | 1 | 0 | 0 |
| Oe_mir_314 | FKYM01005945.1_5994:6021 | + | GTCAATCGAAGCCTCAGTGTTCCCAGAT | | 0 | 3 | 0 | 0 | 0 | 1 | 0 | 0 |
| Oe_mir_315 | FKYM01005943.1_45544:45572 | - | | CCAGTTCCCCGAGGGCCTCCTCATGTACT | 0 | 3 | 0 | 0 | 0 | 1 | 0 | 0 |
| Oe_mir_316 | FKYM01005859.1_33696:33725 | + | | CGTCTTGATTGGAAGCACATGTCTACACTT | 0 | 5 | 0 | 0 | 0 | 1 | 0 | 0 |
| Oe_mir_317 | FKYM01005528.1_1435:1463 | + | | CGCTGCGGATAAGGACAAAGGCACCAAAT | 0 | 3 | 0 | 0 | 0 | 1 | 0 | 0 |

| | | | | | | | | | | | | |
|---|---|---|---|---|---|---|---|---|---|---|---|---|
| Oe_mir_318 | FKYM01005233.1_459:487 | + | TTCAGTCGCCTCCAGTAAATCAATGGGAA | | 0 | 3 | 0 | 0 | 0 | 1 | 0 | 0 |
| Oe_mir_319 | FKYM01005150.1_42797:42825 | - | CTGTCAGGGTTGGCTTACTTGCACAGCAA | | 0 | 3 | 0 | 0 | 0 | 1 | 0 | 0 |
| Oe_mir_320 | FKYM01004445.1_30721:30748 | + | TTATGATCTTGAAGGCAAGACCGTTGGA | | 0 | 4 | 0 | 0 | 0 | 1 | 0 | 0 |
| Oe_mir_321 | FKYM01004346.1_45994:46022 | - | CTCCATACTGCAAAGGGCTGCTGAGAAGA | | 0 | 3 | 0 | 0 | 0 | 1 | 0 | 0 |
| Oe_mir_322 | FKYM01004241.1_35582:35609 | - | CAGAAGTTTTACGTGGAAATGCAAAAGA | | 0 | 6 | 0 | 0 | 0 | 1 | 0 | 0 |
| Oe_mir_323 | FKYM01004020.1_12618:12647 | + | TGTGATAATGGAACTGGAATGGTGAAGGTT | | 0 | 4 | 0 | 0 | 0 | 1 | 0 | 0 |
| Oe_mir_324 | FKYM01003810.1_57390:57417 | + | CGCTGAGATTCAGCCCTTTGTCGCTTCA | | 0 | 4 | 0 | 4 | 0 | 1 | 0 | 1 |
| Oe_mir_325 | FKYM01003810.1_125141:125169 | + | | ATAGTGTACATGTAGCAAGGTCCCAACCG | 0 | 4 | 0 | 0 | 0 | 1 | 0 | 0 |
| Oe_mir_326 | FKYM01003456.1_62278:62305 | - | | AACAGTAGTAATGAACAGTAACAGAAGA | 0 | 3 | 0 | 0 | 0 | 1 | 0 | 0 |
| Oe_mir_327 | FKYM01003264.1_942:972 | + | | TTTGCATGTTTGCACGGATTCGAGGGCCCAA | 0 | 6 | 0 | 0 | 0 | 1 | 0 | 0 |
| Oe_mir_328 | FKYM01003182.1_22696:22723 | - | | CAGTGGCAACGGAATCTAATGGATCCTT | 0 | 4 | 0 | 0 | 0 | 1 | 0 | 0 |
| Oe_mir_329 | FKYM01002918.1_16521:16552 | - | TATACCCAGAGAGAGAGAGAGAGAGAGAGGGA | | 0 | 3 | 0 | 0 | 0 | 1 | 0 | 0 |
| Oe_mir_330 | FKYM01002789.1_15586:15616 | + | | CCATTGCCTGGTTCCGAGCCTCGGACTTCTA | 0 | 3 | 0 | 0 | 0 | 1 | 0 | 0 |
| Oe_mir_331 | FKYM01002677.1_29530:29557 | - | | CAAGACCGCTGAATTTTGATACCGTTGG | 0 | 4 | 0 | 0 | 0 | 1 | 0 | 0 |
| Oe_mir_332 | FKYM01002526.1_49982:50013 | + | CTGTCATCGCTGTTTGATTTATCTGGGGGATC | | 0 | 3 | 0 | 0 | 0 | 1 | 0 | 0 |
| Oe_mir_333 | FKYM01002507.1_947:976 | + | TTGGAGGAATTGTGGGACTAGGCCTGGGAA | | 0 | 4 | 0 | 0 | 0 | 1 | 0 | 0 |
| Oe_mir_334 | FKYM01002091.1_17328:17356 | - | CCCACTGTTCACCAAAACTGTCATACTGT | | 0 | 4 | 0 | 0 | 0 | 1 | 0 | 0 |
| Oe_mir_335 | FKYM01002017.1_7876:7903 | + | | CGTGCTGGTACGGACACTGAGGTTTTAT | 0 | 3 | 0 | 7 | 0 | 1 | 0 | 2 |
| Oe_mir_336 | FKYM01001997.1_45585:45612 | + | TGGTGTTAAGAGGCAAGAAAGTGGAGAT | | 0 | 3 | 0 | 0 | 0 | 1 | 0 | 0 |
| Oe_mir_337 | FKYM01001859.1_141973:142000 | - | | GAGGAACCTCCTGCAGCTGATGATGTGA | 0 | 3 | 0 | 0 | 0 | 1 | 0 | 0 |
| Oe_mir_338 | FKYM01001075.1_1073:1101 | - | CCAAGTCAAGATCTCCCGTCGGTTATCGT | | 0 | 3 | 0 | 0 | 0 | 1 | 0 | 0 |
| Oe_mir_339 | FKYM01000829.1_14769:14800 | + | | TCTTCTGGATTGAGCTCATCTGAAACTAAGCT | 0 | 4 | 0 | 0 | 0 | 1 | 0 | 0 |
| Oe_mir_340 | FKYM01000423.1_115924:115951 | - | TGTTACCTGGACTTGCCTGCCATCATCC | | 0 | 4 | 0 | 0 | 0 | 1 | 0 | 0 |
| Oe_mir_341 | FKYM01000307.1_2869:2899 | + | CTCACTGTAGTACCATTGGAAGAATACTTGG | | 0 | 18 | 0 | 0 | 0 | 3 | 0 | 0 |
| Oe_mir_342 | FKYM01000306.1_55757:55785 | - | CAACAAGAATGTTGAGTACTGGCAGCAGG | | 0 | 3 | 0 | 0 | 0 | 1 | 0 | 0 |
| Oe_mir_343 | FKYM01000034.1_132491:132518 | - | CGAAGTTGATATTATTTCATCATAGCTG | | 0 | 3 | 0 | 0 | 0 | 1 | 0 | 0 |
| Oe_mir_344 | FKYM01056917.1_4069:4096 | + | | GGGTGAACTTGATAAAGACTGATAAACT | 0 | 0 | 3 | 0 | 0 | 0 | 1 | 0 |
| Oe_mir_345 | FKYM01056571.1_23321:23350 | + | CGGTGATATGACCTCTTCTTCTGAGGCAGT | | 0 | 0 | 4 | 0 | 0 | 0 | 2 | 0 |
| Oe_mir_346 | FKYM01055655.1_115505:115533 | + | | TCTACCCTTCGACCACCTTAGCCGTTGGA | 0 | 0 | 3 | 0 | 0 | 0 | 1 | 0 |
| Oe_mir_347 | FKYM01055504.1_37807:37834 | - | | CATTGGGACATGATGTCGTCTGATCCAC | 0 | 0 | 3 | 0 | 0 | 0 | 1 | 0 |
| Oe_mir_348 | FKYM01055053.1_33715:33742 | - | | TTCTTTGACATGACAATCGGCGGTCAAC | 0 | 0 | 3 | 0 | 0 | 0 | 1 | 0 |
| Oe_mir_349 | FKYM01054928.1_4030:4057 | - | | ACTTGGTAGGAATGTAGTATTCTGTTTC | 0 | 0 | 3 | 5 | 0 | 0 | 1 | 2 |

| | | | | | | | | | | | | |
|---|---|---|---|---|---|---|---|---|---|---|---|---|
| Oe_mir_350 | FKYM01054349.1_17122:17150 | - | CTCTGTAGTGGATTGAGTGTGCATCCTTT | | 0 | 0 | 21 | 0 | 0 | 0 | 7 | 0 |
| Oe_mir_351 | FKYM01053416.1_1393:1420 | + | | TAGTGGTATGATTCTCGCTTAGGGTGCG | 0 | 0 | 6 | 0 | 0 | 0 | 2 | 0 |
| Oe_mir_352 | FKYM01052820.1_9440:9468 | + | | CGTGGTAGAAACAACCACACCTCTGACTT | 0 | 0 | 3 | 0 | 0 | 0 | 1 | 0 |
| Oe_mir_353 | FKYM01052084.1_102596:102623 | - | CGAGAATGGAGCTAAGGGTTGTGAGGTA | | 0 | 0 | 3 | 0 | 0 | 0 | 1 | 0 |
| Oe_mir_354 | FKYM01052049.1_2914:2945 | + | | TGGCTGATTAGGAATCCTCTTGAATTTAGGTC | 0 | 0 | 4 | 0 | 0 | 0 | 2 | 0 |
| Oe_mir_355 | FKYM01052049.1_3096:3127 | + | CGTGAGGATTTCCTCTCCGTCAGTATTCTCAT | | 0 | 0 | 1 | 0 | 0 | 0 | 1 | 0 |
| Oe_mir_356 | FKYM01051315.1_42025:42054 | + | GGAGGAGATGAGCCTGAGTAAATCAAAGTG | | 0 | 0 | 3 | 0 | 0 | 0 | 1 | 0 |
| Oe_mir_357 | FKYM01051081.1_22229:22258 | - | | CTTCAAGGAATCCCATGAAGTCCCATGATC | 0 | 0 | 4 | 0 | 0 | 0 | 2 | 0 |
| Oe_mir_358 | FKYM01048149.1_20186:20214 | - | | CATTGAGAGACTCCGTGTTGTTTGACATC | 0 | 0 | 3 | 0 | 0 | 0 | 1 | 0 |
| Oe_mir_359 | FKYM01047269.1_37537:37565 | - | TTGATATATTTGATTACATTAAGAGAATG | | 0 | 0 | 3 | 0 | 0 | 0 | 1 | 0 |
| Oe_mir_360 | FKYM01047076.1_60339:60370 | + | | ATTTTCGCGACTGTTGGAGTGTTGTTAGTTAT | 0 | 0 | 3 | 0 | 0 | 0 | 1 | 0 |
| Oe_mir_361 | FKYM01046746.1_51420:51447 | + | | ATGGACATGAACTTGGAGATGAAGGTGA | 0 | 0 | 3 | 0 | 0 | 0 | 1 | 0 |
| Oe_mir_362 | FKYM01046385.1_14681:14712 | + | | CCCAGTCTCAAGTGCAACCTGACCCTATCATC | 0 | 0 | 9 | 7 | 0 | 0 | 3 | 2 |
| Oe_mir_363 | FKYM01046256.1_64432:64462 | - | CCTTCGGACCCTTGACATTTCATCGCCTTAC | | 0 | 0 | 9 | 0 | 0 | 0 | 3 | 0 |
| Oe_mir_364 | FKYM01044424.1_72272:72299 | + | | CTATGATGTTGATCTCAAATTTGTGCCA | 0 | 0 | 3 | 0 | 0 | 0 | 1 | 0 |
| Oe_mir_365 | FKYM01042757.1_1085:1113 | - | AGGATGAAAATGGTGGAGAGCAGTGAAGA | | 0 | 0 | 3 | 0 | 0 | 0 | 1 | 0 |
| Oe_mir_366 | FKYM01041975.1_2987:3014 | - | GGAGGTGGTCTTAATGAAAAACAAAGCG | | 0 | 0 | 3 | 0 | 0 | 0 | 1 | 0 |
| Oe_mir_367 | FKYM01041563.1_53782:53810 | - | TGAGAAGTCCACTGAACCTTATCATTTAG | | 0 | 0 | 3 | 0 | 0 | 0 | 1 | 0 |
| Oe_mir_368 | FKYM01041531.1_9292:9319 | + | | ACTGAACTCTGTTCTTGAAATTGTGTCT | 0 | 0 | 4 | 0 | 0 | 0 | 2 | 0 |
| Oe_mir_369 | FKYM01041155.1_13199:13226 | + | GGATACTCTGAGATAGAGCGAGAAAAAT | | 0 | 0 | 8 | 0 | 0 | 0 | 3 | 0 |
| Oe_mir_370 | FKYM01040723.1_27723:27753 | - | | CGGGTTCGAATCTTTGGTTGGCACTTCCTTT | 0 | 0 | 4 | 0 | 0 | 0 | 2 | 0 |
| Oe_mir_371 | FKYM01040102.1_14957:14985 | + | CTCCCCAAATTAGCCTTGCCCAGTATCCC | | 0 | 0 | 3 | 0 | 0 | 0 | 1 | 0 |
| Oe_mir_372 | FKYM01038735.1_2416:2443 | - | CTTGGTTACAAGTAGATAGTGGCTAGCT | | 0 | 0 | 3 | 0 | 0 | 0 | 1 | 0 |
| Oe_mir_373 | FKYM01035905.1_70113:70141 | - | | CAGTTGGTTAGAGCGTGTGGCTGTTAACC | 0 | 0 | 5 | 6 | 0 | 0 | 2 | 2 |
| Oe_mir_374 | FKYM01035436.1_188744:188772 | + | | GTGGCCAAGTGGTAAGGCAACGGGTTTTG | 0 | 0 | 5 | 10 | 0 | 0 | 2 | 3 |
| Oe_mir_375 | FKYM01034996.1_25393:25420 | + | ATACTCTGAGGATGTTCTGCATTGGCTT | | 0 | 0 | 3 | 0 | 0 | 0 | 1 | 0 |
| Oe_mir_376 | FKYM01034384.1_70207:70234 | + | TCTCTGAGCACCCGATGTCTGCTACTAT | | 0 | 0 | 3 | 0 | 0 | 0 | 1 | 0 |
| Oe_mir_377 | FKYM01034242.1_1585:1612 | + | ATAGGTTTAATTTCCTGTAGACTCACAT | | 0 | 0 | 5 | 0 | 0 | 0 | 2 | 0 |
| Oe_mir_378 | FKYM01034207.1_114302:114330 | - | TTACTCCGAGGCATCTCCAGCTTGCTATT | | 0 | 0 | 3 | 0 | 0 | 0 | 1 | 0 |
| Oe_mir_379 | FKYM01033615.1_75822:75849 | - | CTGCCACTTGTTGATTTTGTAACCCATT | | 0 | 0 | 3 | 0 | 0 | 0 | 1 | 0 |
| Oe_mir_380 | FKYM01033607.1_64785:64814 | - | | AGACAAAGATTTGAGCCCTAAGATTTCAGA | 0 | 0 | 3 | 0 | 0 | 0 | 1 | 0 |
| Oe_mir_381 | FKYM01032996.1_78923:78950 | + | | GAACTTGACAGTCCTCCTCACAAATCGC | 0 | 0 | 3 | 0 | 0 | 0 | 1 | 0 |

| | | | | | | | | | | | |
|---|---|---|---|---|---|---|---|---|---|---|---|
| Oe_mir_382 | FKYM01032621.1_5808:5838 | - | | TTGGTTAAAGGCTTGTTTGGATTCATATAGT | 0 | 0 | 3 | 0 | 0 | 0 | 1 | 0 |
| Oe_mir_383 | FKYM01032104.1_25853:25882 | + | TGGATCAAGGTCTGGATTATGTCGCCACAT | | 0 | 0 | 8 | 0 | 0 | 0 | 3 | 0 |
| Oe_mir_384 | FKYM01031755.1_83362:83390 | + | | AATGGCAAGAGTAGGCGGGCACCGATACT | 0 | 0 | 6 | 0 | 0 | 0 | 2 | 0 |
| Oe_mir_385 | FKYM01030426.1_12797:12825 | - | | TGGAAGGGACGCATTTATTAGATAAATGG | 0 | 0 | 4 | 0 | 0 | 0 | 2 | 0 |
| Oe_mir_386 | FKYM01030409.1_2386:2414 | + | ATAGTTACGTGCGGATGGTTGCATCTATT | | 0 | 0 | 3 | 0 | 0 | 0 | 1 | 0 |
| Oe_mir_387 | FKYM01029890.1_12727:12756 | - | GATGTCGATCCTGAGCGGTTACCTGAGCAA | | 0 | 0 | 57 | 0 | 0 | 0 | 19 | 0 |
| Oe_mir_388 | FKYM01029816.1_35171:35198 | - | | TGATTTTTATGCAAGCTGCTAAGTCCGT | 0 | 0 | 3 | 0 | 0 | 0 | 1 | 0 |
| Oe_mir_389 | FKYM01028429.1_562:589 | + | | CATCGACTCCGATCTGTATTGTCTCGTT | 0 | 0 | 3 | 0 | 0 | 0 | 1 | 0 |
| Oe_mir_390 | FKYM01027163.1_47489:47518 | + | | GTCCCGTGATTGGTGTTCGTGCCAAGCTGA | 0 | 0 | 3 | 0 | 0 | 0 | 1 | 0 |
| Oe_mir_391 | FKYM01025940.1_24229:24256 | + | GCGATTTCCTTGGCTACGGAGTGTGTTC | | 0 | 0 | 3 | 0 | 0 | 0 | 1 | 0 |
| Oe_mir_392 | FKYM01025563.1_59213:59241 | + | CTTTGTCTTTGGGGCTTACAATTCACCTA | | 0 | 0 | 5 | 22 | 0 | 0 | 2 | 6 |
| Oe_mir_393 | FKYM01025050.1_77586:77615 | + | GTGCTTGTGAACCCTTGCTCTCTTGAAGCC | | 0 | 0 | 3 | 0 | 0 | 0 | 1 | 0 |
| Oe_mir_394 | FKYM01024844.1_50311:50339 | - | | TCGGTGGTGATGAAGTCGAAAACGGAGTG | 0 | 0 | 3 | 0 | 0 | 0 | 1 | 0 |
| Oe_mir_395 | FKYM01023070.1_36911:36938 | - | | CTTGGACCGTTGGATTGATCCGCTGATC | 0 | 0 | 3 | 0 | 0 | 0 | 1 | 0 |
| Oe_mir_396 | FKYM01022924.1_253:282 | + | | AACGTCGAGGAACATGTACTAGGGTGTATG | 0 | 0 | 4 | 0 | 0 | 0 | 2 | 0 |
| Oe_mir_397 | FKYM01022360.1_18268:18298 | - | GCCCTGGGAAGTCCTCGTGTTGCACCCCTTT | | 0 | 0 | 73 | 0 | 0 | 0 | 24 | 0 |
| Oe_mir_398 | FKYM01022359.1_9984:10015 | - | GGGTGACCCCCTGGGAAGTCCTCGTGTTGCCC | | 0 | 0 | 7 | 0 | 0 | 0 | 3 | 0 |
| Oe_mir_399 | FKYM01022358.1_11341:11368 | - | | CACCTGGGAAGTCCTCGTGTTGCCCCCT | 0 | 0 | 4 | 0 | 0 | 0 | 2 | 0 |
| Oe_mir_400 | FKYM01022358.1_11011:11039 | - | CCTCCTGGGAAGTCCTCGTGTTGCACCCC | | 0 | 0 | 11 | 0 | 0 | 0 | 4 | 0 |
| Oe_mir_401 | FKYM01022014.1_1538:1568 | + | | TCTGTGATGGTAAATGGGTGTGGTCTGAGCC | 0 | 0 | 4 | 0 | 0 | 0 | 2 | 0 |
| Oe_mir_402 | FKYM01021782.1_22601:22628 | + | TTCCATCACGTCTTTGTTGTAGGGCAGG | | 0 | 0 | 8 | 9 | 0 | 0 | 3 | 3 |
| Oe_mir_403 | FKYM01020958.1_18271:18300 | + | | GCATTTGGTCTAGTGGTATGATTCTCGCTT | 0 | 0 | 21 | 0 | 0 | 0 | 7 | 0 |
| Oe_mir_404 | FKYM01019895.1_7600:7630 | - | | CTTTATTACGTCTGGGAGCTGGAGCTTGTGC | 0 | 0 | 4 | 0 | 0 | 0 | 2 | 0 |
| Oe_mir_405 | FKYM01018308.1_24987:25016 | + | TTTCAGCAACGGATTTCTTGGCTCTCGCAT | | 0 | 0 | 5 | 0 | 0 | 0 | 2 | 0 |
| Oe_mir_406 | FKYM01017665.1_47362:47391 | - | ATAAATGTTGGGCTGTTTATTTCTTGTCAC | | 0 | 0 | 4 | 0 | 0 | 0 | 2 | 0 |
| Oe_mir_407 | FKYM01015766.1_8152:8179 | + | | TGGCAGCTGGAGAACTTGAAAACGCAAT | 0 | 0 | 3 | 0 | 0 | 0 | 1 | 0 |
| Oe_mir_408 | FKYM01015060.1_2770:2799 | + | GGATCTGAGAAAAGCTCCAGTGCTGAAGGA | | 0 | 0 | 4 | 0 | 0 | 0 | 2 | 0 |
| Oe_mir_409 | FKYM01014142.1_11310:11340 | - | | CACTGCCAGAGAACTTGTGGAGCAACCCATA | 0 | 0 | 3 | 0 | 0 | 0 | 1 | 0 |
| Oe_mir_410 | FKYM01013352.1_54315:54343 | - | GCTGTCTATCATCCTGAATTCGGTTTAGA | | 0 | 0 | 4 | 6 | 0 | 0 | 2 | 2 |
| Oe_mir_411 | FKYM01013088.1_48462:48490 | + | GGGAGGAGCAACTGAAGGAACAGATGGCT | | 0 | 0 | 3 | 0 | 0 | 0 | 1 | 0 |
| Oe_mir_412 | FKYM01012168.1_18221:18252 | - | | CAACGAAGACTGCAGTGTAGCTGAGCCTATAG | 0 | 0 | 3 | 0 | 0 | 0 | 1 | 0 |
| Oe_mir_413 | FKYM01011530.1_43070:43097 | + | | GTTGGTCTTCAACGAGGAATTCCTAGTA | 0 | 0 | 3 | 0 | 0 | 0 | 1 | 0 |

| | | | | | | | | | | | | |
|---|---|---|---|---|---|---|---|---|---|---|---|---|
| Oe_mir_414 | FKYM01010415.1_37449:37477 | + | | GTTTGACAAACTCGACACTTGGCGGCCCC | 0 | 0 | 3 | 0 | 0 | 0 | 1 | 0 |
| Oe_mir_415 | FKYM01007418.1_15487:15515 | - | | CCGTGTTTATGGATTGGATCGTTTCTGCC | 0 | 0 | 7 | 0 | 0 | 0 | 3 | 0 |
| Oe_mir_416 | FKYM01006167.1_127537:127566 | - | CAAGAAGAGGAATGTAAAACTTGTGCCATG | | 0 | 0 | 3 | 0 | 0 | 0 | 1 | 0 |
| Oe_mir_417 | FKYM01004872.1_28641:28669 | - | CAGAGAACTTACAATAATACAACGCCGCA | | 0 | 0 | 3 | 0 | 0 | 0 | 1 | 0 |
| Oe_mir_418 | FKYM01004493.1_7151:7181 | - | | CTCCTCCTCTCACATGGGGGTCGGACTCCAC | 0 | 0 | 3 | 0 | 0 | 0 | 1 | 0 |
| Oe_mir_419 | FKYM01003996.1_54815:54844 | - | CTGGTTGCTCTGGTTTATTTGGTGTCGTTT | | 0 | 0 | 3 | 0 | 0 | 0 | 1 | 0 |
| Oe_mir_420 | FKYM01003711.1_24032:24059 | + | | CCTACCTTATTGACCGCAACTTCTGTAT | 0 | 0 | 21 | 0 | 0 | 0 | 7 | 0 |
| Oe_mir_421 | FKYM01002812.1_8506:8533 | + | | TTTTGGTCTTTCAAGGGTGTTCATTGTC | 0 | 0 | 5 | 0 | 0 | 0 | 2 | 0 |
| Oe_mir_422 | FKYM01002408.1_65648:65677 | + | AGTGTGAAAATCCAATGGTGTGGGCTCCTG | | 0 | 0 | 4 | 0 | 0 | 0 | 2 | 0 |
| Oe_mir_423 | FKYM01001628.1_127866:127894 | + | | TCACATCGACGGGAAGGTTTGGCACCTCG | 0 | 0 | 3 | 0 | 0 | 0 | 1 | 0 |
| Oe_mir_424 | FKYM01001537.1_46654:46681 | + | | AGAGTTAACTATTTGTATGTCGTGTAGA | 0 | 0 | 4 | 0 | 0 | 0 | 2 | 0 |
| Oe_mir_425 | FKYM01000034.1_133738:133766 | - | CTCATCGGCGGTGTCCATGCAGATTGGCG | | 0 | 0 | 3 | 0 | 0 | 0 | 1 | 0 |
| Oe_mir_426 | FKYM01055738.1_692:719 | - | | CGGTGCATCGCAAACTCTATTTAGTAAG | 0 | 0 | 0 | 4 | 0 | 0 | 0 | 1 |
| Oe_mir_427 | FKYM01055253.1_22607:22638 | + | | CGGGTTCGAATCTTTGGTTGGCACTTCCTTAG | 0 | 0 | 0 | 7 | 0 | 0 | 0 | 2 |
| Oe_mir_428 | FKYM01054914.1_2650:2677 | + | CTTTTCGTTGAGTTGGGTCGGGCAAGGA | | 0 | 0 | 0 | 4 | 0 | 0 | 0 | 1 |
| Oe_mir_429 | FKYM01054349.1_17121:17150 | - | CTCTGTAGTGGATTGAGTGTGCATCCTTTT | | 0 | 0 | 0 | 6 | 0 | 0 | 0 | 2 |
| Oe_mir_430 | FKYM01053846.1_27861:27890 | + | | CACCACCATTTTCACAACCTATCAAACTGC | 0 | 0 | 0 | 3 | 0 | 0 | 0 | 1 |
| Oe_mir_431 | FKYM01053416.1_1715:1746 | - | | CATTTGGTCTAGTGGTATGATTCTCGCTTTGG | 0 | 0 | 0 | 3 | 0 | 0 | 0 | 1 |
| Oe_mir_432 | FKYM01052374.1_12957:12987 | - | GGTGCTTGTTTCGCTTGAATAAAAATACAGT | | 0 | 0 | 0 | 3 | 0 | 0 | 0 | 1 |
| Oe_mir_433 | FKYM01052158.1_37789:37817 | - | | TCCCATAACTCCAAAGGATTCGGTCGGAA | 0 | 0 | 0 | 4 | 0 | 0 | 0 | 1 |
| Oe_mir_434 | FKYM01052107.1_7040:7067 | - | CTCGGACCAGGCTTCATTCCCCCCAACT | | 0 | 0 | 0 | 3 | 0 | 0 | 0 | 1 |
| Oe_mir_435 | FKYM01051969.1_19399:19427 | + | | TCAAGGTGATGCTGATGATAAACTGGTGC | 0 | 0 | 0 | 3 | 0 | 0 | 0 | 1 |
| Oe_mir_436 | FKYM01051778.1_52:81 | - | CAAGGCTCTTAACCTTGTGGTCGTGGGTTC | | 0 | 0 | 0 | 3 | 0 | 0 | 0 | 1 |
| Oe_mir_437 | FKYM01050294.1_1154:1182 | - | CTCTGAGTTCGACGTTGAATAGCCATAGT | | 0 | 0 | 0 | 4 | 0 | 0 | 0 | 1 |
| Oe_mir_438 | FKYM01047025.1_8816:8844 | + | TACTTGTGAAAACTGAATGCAAGATGTTT | | 0 | 0 | 0 | 3 | 0 | 0 | 0 | 1 |
| Oe_mir_439 | FKYM01046256.1_64435:64464 | - | CTCCTTCGGACCCTTGACATTTCATCGCCT | | 0 | 0 | 0 | 11 | 0 | 0 | 0 | 3 |
| Oe_mir_440 | FKYM01046040.1_6951:6979 | - | CAGAGGTCGTGAGTTCGAAACATGGGGCT | | 0 | 0 | 0 | 3 | 0 | 0 | 0 | 1 |
| Oe_mir_441 | FKYM01044089.1_10441:10469 | + | | GTGTGATTAAAAATATGAAAGAGAAAAAA | 0 | 0 | 0 | 3 | 0 | 0 | 0 | 1 |
| Oe_mir_442 | FKYM01043838.1_1480:1507 | + | | TCGTTCATTTCCATTTCCATCGACCATT | 0 | 0 | 0 | 4 | 0 | 0 | 0 | 1 |
| Oe_mir_443 | FKYM01042756.1_3717:3745 | - | | TCTTAACCACTGCTCCACCTTGGAATCAC | 0 | 0 | 0 | 3 | 0 | 0 | 0 | 1 |
| Oe_mir_444 | FKYM01042675.1_28157:28185 | + | CGAGGAGGGGCTCATGACGGGGGTTGTGG | | 0 | 0 | 0 | 4 | 0 | 0 | 0 | 1 |
| Oe_mir_445 | FKYM01042141.1_13034:13062 | + | | CCAACATGTCAGGAAATAAGAGCTTGCTC | 0 | 0 | 0 | 3 | 0 | 0 | 0 | 1 |

| | | | | | | | | | | | | |
|---|---|---|---|---|---|---|---|---|---|---|---|---|
| Oe_mir_446 | FKYM01041563.1_53968:53996 | - | | TGTGATGGGGATAGATCATTGCAATTGTT | 0 | 0 | 0 | 3 | 0 | 0 | 0 | 1 |
| Oe_mir_447 | FKYM01041226.1_24538:24566 | + | ACCCATGGGGATTCACAAGTCTGAGATGC | | 0 | 0 | 0 | 14 | 0 | 0 | 0 | 4 |
| Oe_mir_448 | FKYM01040730.1_160344:160373 | - | CGCTCGGCTCGCCGTTGACTCTGGTGGTGA | | 0 | 0 | 0 | 3 | 0 | 0 | 0 | 1 |
| Oe_mir_449 | FKYM01040669.1_64843:64874 | - | | CGGACGAGAAAGACAGTGCTGACGGTGGAGAG | 0 | 0 | 0 | 3 | 0 | 0 | 0 | 1 |
| Oe_mir_450 | FKYM01039618.1_3599:3629 | - | CTTCCAGCAATTTATAATAATCCAGACGTAC | | 0 | 0 | 0 | 3 | 0 | 0 | 0 | 1 |
| Oe_mir_451 | FKYM01038484.1_2158:2187 | + | | GTTGTCCCTTCTCGTTCTCTATTATTATCT | 0 | 0 | 0 | 3 | 0 | 0 | 0 | 1 |
| Oe_mir_452 | FKYM01038462.1_1224:1252 | + | TGGTTGATCCTGCCAGTAGTCATATGCTT | | 0 | 0 | 0 | 10 | 0 | 0 | 0 | 3 |
| Oe_mir_453 | FKYM01036119.1_83981:84012 | - | | CCGGGTTCGAATCTTTGGTTGGCACTTCCTTT | 0 | 0 | 0 | 62 | 0 | 0 | 0 | 16 |
| Oe_mir_454 | FKYM01035439.1_6502:6533 | - | | TTGTTGTCGATGTTGTGGTAAACTGAGCCCGA | 0 | 0 | 0 | 5 | 0 | 0 | 0 | 2 |
| Oe_mir_455 | FKYM01034081.1_28300:28331 | - | CTTTGGAAATTTAGGCTCTGTGTTTGTTTGGG | | 0 | 0 | 0 | 3 | 0 | 0 | 0 | 1 |
| Oe_mir_456 | FKYM01033960.1_62696:62723 | + | CGAACCCTTGACGCGGCAGCCTCAATCA | | 0 | 0 | 0 | 3 | 0 | 0 | 0 | 1 |
| Oe_mir_457 | FKYM01033524.1_70787:70817 | - | CTGAATGATCTCGGACCAGGCTTCATTCCCC | | 0 | 0 | 0 | 3 | 0 | 0 | 0 | 1 |
| Oe_mir_458 | FKYM01032968.1_33117:33144 | - | GTGGCACTTACTTACTTGTATAGAGATA | | 0 | 0 | 0 | 5 | 0 | 0 | 0 | 2 |
| Oe_mir_459 | FKYM01032634.1_9323:9351 | + | ACTCCCTGTTGTAATAATGTTTAGATCAT | | 0 | 0 | 0 | 3 | 0 | 0 | 0 | 1 |
| Oe_mir_460 | FKYM01031741.1_33313:33343 | + | | TTTGGGTGCGAGAGGTCCCGAGTTCGATTCT | 0 | 0 | 0 | 3 | 0 | 0 | 0 | 1 |
| Oe_mir_461 | FKYM01031732.1_27776:27803 | - | | TAGACCTTGTTGTTGTCAGAATTCTTAA | 0 | 0 | 0 | 10 | 0 | 0 | 0 | 3 |
| Oe_mir_462 | FKYM01030017.1_17868:17897 | + | | TTGGCGAGGAAGGTGTCTCTGTTTAGGGCG | 0 | 0 | 0 | 3 | 0 | 0 | 0 | 1 |
| Oe_mir_463 | FKYM01030017.1_18270:18299 | + | TGGAACTGGTATAAACCCTGCTAGGAGCTT | | 0 | 0 | 0 | 2 | 0 | 0 | 0 | 1 |
| Oe_mir_464 | FKYM01027470.1_5567:5595 | - | CTCATGGCCTAGTATTTCACATCGTTAAC | | 0 | 0 | 0 | 3 | 0 | 0 | 0 | 1 |
| Oe_mir_465 | FKYM01026946.1_9147:9175 | - | | TACATGGAAATACTCAGTAGAATATAAAA | 0 | 0 | 0 | 3 | 0 | 0 | 0 | 1 |
| Oe_mir_466 | FKYM01025050.1_61504:61532 | + | | AAGTCTCAGGATCGTATTATTGGAGCCCA | 0 | 0 | 0 | 3 | 0 | 0 | 0 | 1 |
| Oe_mir_467 | FKYM01025050.1_77573:77604 | + | CTCTGGCTGTCGCGTGCTTGTGAACCCTTGCT | | 0 | 0 | 0 | 3 | 0 | 0 | 0 | 1 |
| Oe_mir_468 | FKYM01025035.1_62024:62052 | + | CCCTAATCGGCTTGTTGTAGACGAGGCCG | | 0 | 0 | 0 | 3 | 0 | 0 | 0 | 1 |
| Oe_mir_469 | FKYM01024763.1_628:656 | - | GGGGATGCCGAAGGCAGGGCTAGTGACTG | | 0 | 0 | 0 | 16 | 0 | 0 | 0 | 4 |
| Oe_mir_470 | FKYM01023719.1_39867:39896 | - | | TGTTGGGGCTGACTACTTTGACAGGCCACA | 0 | 0 | 0 | 3 | 0 | 0 | 0 | 1 |
| Oe_mir_471 | FKYM01022884.1_67927:67955 | - | | CCGAACGCTACGGTCCACTCATGATGCTT | 0 | 0 | 0 | 4 | 0 | 0 | 0 | 1 |
| Oe_mir_472 | FKYM01022360.1_19589:19618 | - | CCCCCTGGGAAGTCCTCGTGTTGTACCTCT | | 0 | 0 | 0 | 6 | 0 | 0 | 0 | 2 |
| Oe_mir_473 | FKYM01022359.1_5334:5362 | - | | CCCATGGGAAGTCCTCGTGTTGCACCCCT | 0 | 0 | 0 | 9 | 0 | 0 | 0 | 3 |
| Oe_mir_474 | FKYM01022358.1_22800:22827 | - | CTTGGGCGAGAGTAGAACTAGGATGGGT | | 0 | 0 | 0 | 3 | 0 | 0 | 0 | 1 |
| Oe_mir_475 | FKYM01021684.1_47380:47409 | - | CCGACAGGAAGGATAGGAATGGCTGCCCCA | | 0 | 0 | 0 | 3 | 0 | 0 | 0 | 1 |
| Oe_mir_476 | FKYM01020992.1_55159:55190 | - | | TAGGCTGGTTCGGATTGGTTATACATAGTTCC | 0 | 0 | 0 | 13 | 0 | 0 | 0 | 4 |
| Oe_mir_477 | FKYM01020978.1_1376:1407 | - | CTAATGAATTCCCTGGGAACTTCTATAGTAAA | | 0 | 0 | 0 | 4 | 0 | 0 | 0 | 1 |

| | | | | | | | | | | | |
|---|---|---|---|---|---|---|---|---|---|---|---|
| Oe_mir_478 | FKYM01014973.1_4534:4561 | - | CGGGCCTGGGTTCACTGGAAGAAGCTAG | | 0 | 0 | 0 | 3 | 0 | 0 | 0 | 1 |
| Oe_mir_479 | FKYM01013831.1_82961:82990 | + | | TTCAATCCAATAGATCTGATCTGAGCCTCA | 0 | 0 | 0 | 14 | 0 | 0 | 0 | 4 |
| Oe_mir_480 | FKYM01012340.1_10912:10941 | + | GGCAGGGGCTATATTAAGAGCAGAGACCTT | | 0 | 0 | 0 | 3 | 0 | 0 | 0 | 1 |
| Oe_mir_481 | FKYM01011433.1_17511:17539 | + | | CCCTATTTGTTTGGTCCGTTCTAGTGATA | 0 | 0 | 0 | 16 | 0 | 0 | 0 | 4 |
| Oe_mir_482 | FKYM01008235.1_32541:32568 | - | | TTTTGTTTGATTTGAACTTTTTGATCAG | 0 | 0 | 0 | 3 | 0 | 0 | 0 | 1 |
| Oe_mir_483 | FKYM01006869.1_3254:3284 | - | | CCCCGTGAAACTCTCCATGAACTTTTGAAAC | 0 | 0 | 0 | 3 | 0 | 0 | 0 | 1 |
| Oe_mir_484 | FKYM01004084.1_58055:58083 | + | | AGTCGACTTTAGCGAAACACCCTTTGACA | 0 | 0 | 0 | 4 | 0 | 0 | 0 | 1 |
| Oe_mir_485 | FKYM01003121.1_36001:36030 | + | AATGGCCTCCACCATCCATCACCGCCGGCT | | 0 | 0 | 0 | 3 | 0 | 0 | 0 | 1 |
| Oe_mir_486 | FKYM01002413.1_7066:7097 | + | TTTCATAGCTCAGTTGGTTAGAGCACCCGTTT | | 0 | 0 | 0 | 4 | 0 | 0 | 0 | 1 |
| Oe_mir_487 | FKYM01002296.1_87009:87040 | + | | ATCGTGGATCGGTGGACAGCATTCCTTTAACC | 0 | 0 | 0 | 3 | 0 | 0 | 0 | 1 |
| Oe_mir_488 | FKYM01002105.1_14741:14770 | - | CTTGAGGATGGCCGCACTCTTGCCGATTAC | | 0 | 0 | 0 | 3 | 0 | 0 | 0 | 1 |
| Oe_mir_489 | FKYM01001537.1_46651:46681 | + | | CTCAGAGTTAACTATTTGTATGTCGTGTAGA | 0 | 0 | 0 | 3 | 0 | 0 | 0 | 1 |
| Oe_mir_490 | FKYM01000977.1_18441:18469 | + | | TGAGACTGGTGTTGGATATGGGGAGTGGT | 0 | 0 | 0 | 3 | 0 | 0 | 0 | 1 |
| Oe_mir_491 | FKYM01000760.1_40439:40467 | + | | GGTTTACCGGCTGTGTACCCCTTGACCCT | 0 | 0 | 0 | 7 | 0 | 0 | 0 | 2 |
| Oe_mir_492 | FKYM01000760.1_40646:40673 | + | GGATTGAATGGACTGAGACCGAGGAACT | | 0 | 0 | 0 | 1 | 0 | 0 | 0 | 1 |
